# Lipid-associated macrophages transition to an inflammatory state in human atherosclerosis, increasing the risk of cerebrovascular complications

**DOI:** 10.1038/s44161-023-00295-x

**Published:** 2023-06-26

**Authors:** Lea Dib, Lada A. Koneva, Andreas Edsfeldt, Yasemin-Xiomara Zurke, Jiangming Sun, Mihaela Nitulescu, Moustafa Attar, Esther Lutgens, Steffen Schmidt, Marie W. Lindholm, Robin P. Choudhury, Ismail Cassimjee, Regent Lee, Ashok Handa, Isabel Goncalves, Stephen N. Sansom, Claudia Monaco

**Affiliations:** 1 Kennedy Institute of Rheumatology, Nuffield Department of Orthopaedics, Rheumatology and Musculoskeletal Sciences, University of Oxford, Oxford, UK; 2 Department of Clinical Sciences Malmö, Clinical Research Center, Lund University, Malmö, Sweden; 3 Department of Cardiology, Skåne University Hospital, Malmö, Sweden; 4 Wallenberg Center for Molecular Medicine, Lund University, Lund, Sweden; 5 Cardiovascular Medicine and Immunology, Mayo Clinic, Rochester, MN USA; 6 Roche Pharma Research and Early Development, RNA Therapeutics Research, Roche Innovation Center Copenhagen, Hørsholm, Denmark; 7 Radcliffe Department of Medicine, University of Oxford, Oxford, UK; 8 Nuffield Department of Surgical Sciences, University of Oxford, Oxford, UK

**Keywords:** Cardiovascular biology, Inflammation

## Abstract

The immune system is integral to cardiovascular health and disease. Targeting inflammation ameliorates adverse cardiovascular outcomes. Atherosclerosis, a major underlying cause of cardiovascular disease, is conceptualized as lipid-driven inflammation in which macrophages play a nonredundant role. However, evidence emerging so far from single-cell atlases suggests a dichotomy between lipid-associated and inflammatory macrophage states. Here, we present an inclusive reference atlas of human intraplaque immune cell communities. Combining single-cell RNA sequencing (scRNA-seq) of human surgical carotid endarterectomies in a discovery cohort with bulk RNA-seq and immunohistochemistry in a validation cohort (the Carotid Plaque Imaging Project), we reveal the existence of PLIN2^hi^/TREM1^hi^ macrophages as a Toll-like receptor (TLR)-dependent inflammatory lipid-associated macrophage state linked to cerebrovascular events. Our study shifts the current paradigm of lipid-driven inflammation by providing biological evidence for a pathogenic macrophage transition to an inflammatory lipid-associated phenotype and for its targeting as a new treatment strategy for cardiovascular disease.

## Abstract (web summary)

Dib, Koneva et al. generate an scRNA-seq atlas of immune cells from human carotid endarterectomies and report the presence of a subset of pathogenic pro-inflammatory PLIN2^hi^/TREM1^hi^, derived from TREM2^hi^ lipid-associated macrophages via TLR signaling, which correlates with an increased risk of cerebrovascular events.

## Main

Atherosclerosis is the underlying pathology in a large majority of cases of myocardial infarction and is a major factor in ischemic stroke. Phase III clinical trials have recently provided evidence that targeting inflammation ameliorates cardiovascular outcomes^1,2^. Evidence has accumulated over several years in support of the concept of atherosclerosis as lipid-driven inflammation. This concept largely centers on the biology of the so-called foam cell, the hallmark of atherosclerosis^3^. Accumulation and retention of cholesterol-rich lipoproteins is a major perpetuating factor of inflammation within the vessel wall^4,5^. Lipid-associated macrophages (LAMs) are not unique to atherosclerosis but are a common denominator of several human diseases of different pathogenesis, including myelin degenerating diseases^6^, nonalcoholic steatohepatitis (NASH)^7^ and obesity^8^. Recent advances in single-cell biology have identified a common LAM state across these diseases, namely the triggering receptor expressed on myeloid cells 2 high (TREM2^hi^) macrophages^9^. The lack of inflammation signatures in TREM2^hi^ macrophages^9–11^ is, however, at odds with the central tenet of atherogenesis as lipid-driven inflammation^12^.

Using single-cell transcriptomics to profile approximately 22,000 CD45^+^ live cells derived from human carotid endarterectomy specimens (‘discovery cohort’), combined with bulk RNA-seq and immunohistochemistry in the Carotid Plaque Imaging Project (CPIP) study (‘validation cohort’), we identify a community of perilipin 2^hi^ (PLIN2^hi^)/TREM1^hi^ plaque macrophages that couples transcriptomic signatures of lipid accumulation and inflammation. Trajectory analysis, ligand–receptor interaction analysis, and histological and functional studies show that intraplaque LAMs transition from a TREM2^hi^ homeostatic to a PLIN2^hi^/TREM1^hi^ inflammatory transcriptional state, and that TLR2 signaling is important in this phenotypic switch. In the CPIP cohort (*n* = 115), the transcriptional and protein signature of inflammatory LAMs is enriched in plaques from patients with carotid artery disease who recently experienced an ischemic cerebrovascular event compared with those who did not. Our study reveals the cellular basis of lipid-driven inflammation in human atherosclerosis and links it to plaque vulnerability to complications, highlighting new avenues for treatment of cardiovascular disease.

## Results

### T cells and mononuclear phagocytes in human carotid plaques

**Extended Data Fig. 1:**
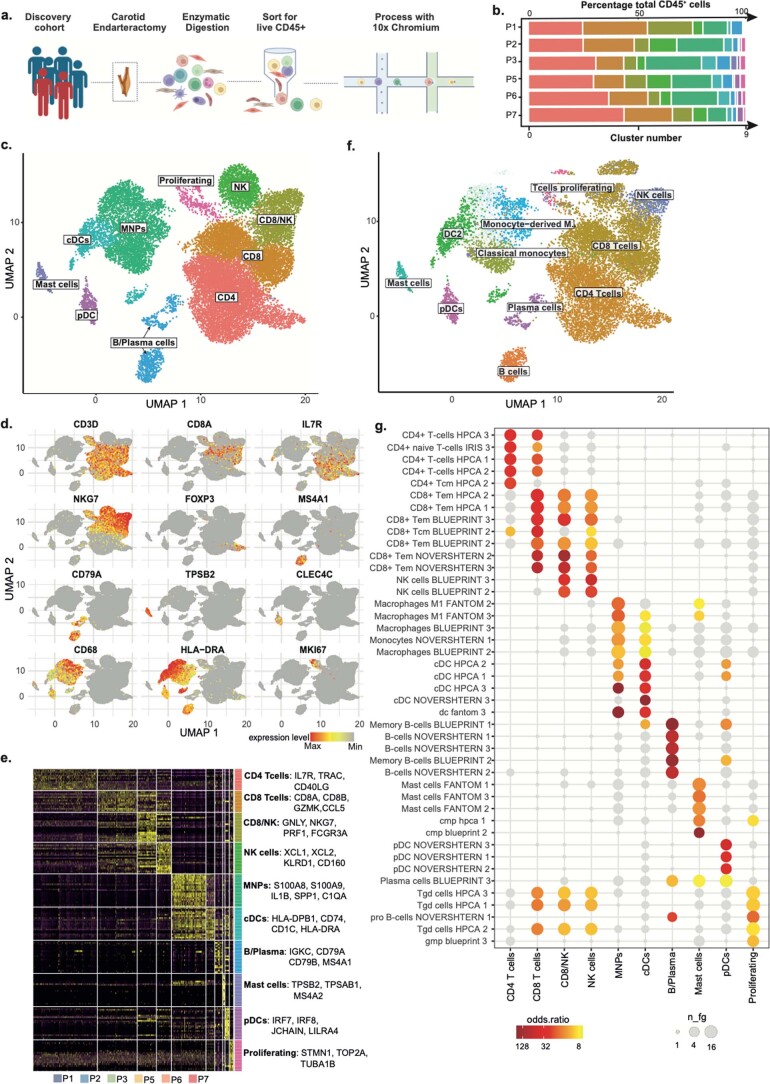
Single-cell RNA sequencing of human atherosclerotic plaque. Atherosclerotic plaques were collected from n = 6 patients undergoing CEA, enzymatically digested, sorted for CD45^+^ cells and subjected to scRNA-seq analysis using 10x Chromium platform (Diagram created by Biorender) **(a)**. The barplots show the frequencies of the clusters distribution in the different patients **(b)**. Unsupervised Leiden clustering of the integrated dataset identified n = 10 distinct clusters encompassing all major immune cell populations **(c)**. The clusters were annotated by classical marker gene expression and by their top marker genes (BH adjusted p < 0.05; Wilcoxon tests) **(d,e)**. Automatic cell type predictions computed using Azimuth^18^ to map cells to the Lung v2 (HLCA)^73–81^ reference data set (**f**). Gene set over-representation analysis of cluster markers using sets of known cell type marker genes from xCell^17^(**g**).

Our discovery cohort consisted of six patients undergoing carotid endarterectomy. Tissues were enzymatically digested, sorted for live CD45^+^ cells and subjected to scRNA-seq. After removing low-quality cells and doublets (see Methods, Supplementary Fig. 1), we retained a total of *n* = 20,943 cells comprising all major immune cell types known to be present in plaques, including T cells, mononuclear phagocytes (MNPs), B cells, plasma cells and mast cells (Extended Data Fig. 1). As previously reported^11^, T cells formed the majority of CD45^+^ cells (mean frequency, 52%), followed by MNPs (mean frequency, 18%) (Extended Data Fig. 1). To investigate the cellular heterogeneity present within the T/natural killer (NK) lymphocyte and MNP cell compartments, we performed more granular analyses of each of these major cell subsets separately using the Scanpy toolkit^13^ and Harmony integration algorithm^14^.

**Fig. 1:**
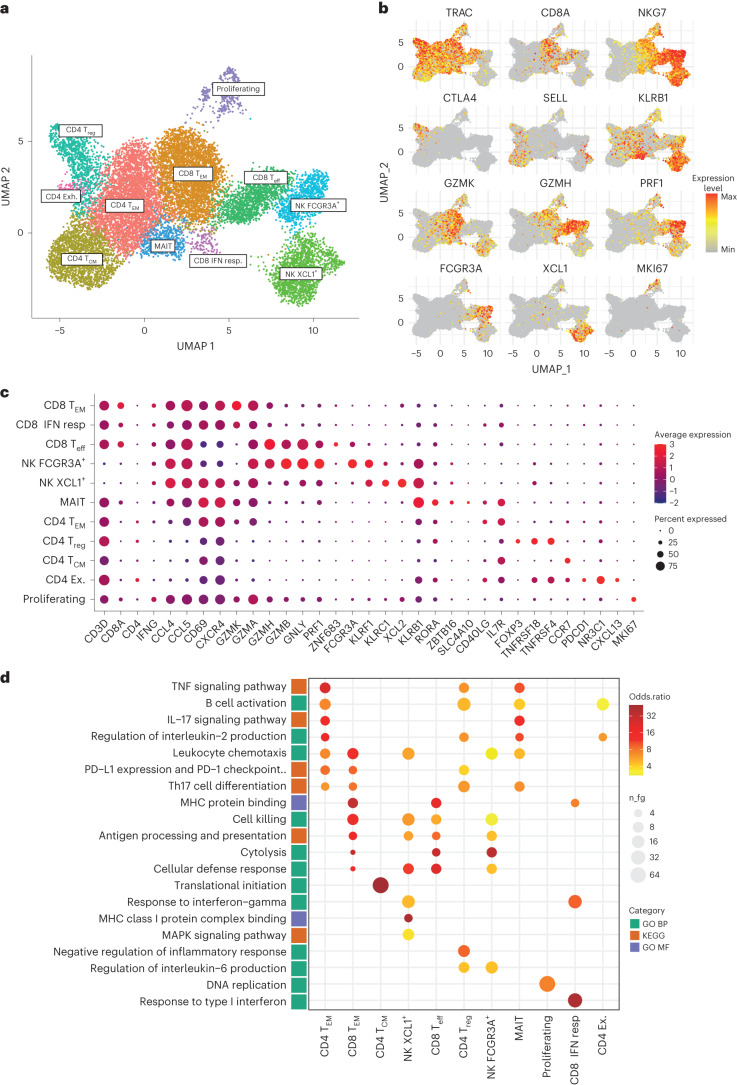
Identification of plaque T and NK cell populations with cytotoxic and activation signatures. **a**–**d**, Cells identified as CD4 T, CD8 T, NK and proliferating cells in the overall analysis (*n* = 15,052 cells) were extracted and analyzed separately. **a**, The UMAP shows the 11 identified lymphocyte subpopulations. **b**,**c**, The expression of selected automatically discovered cluster marker genes (BH-adjusted *P* < 0.05, two-sided Wilcoxon tests) and known cell-type marker genes is shown on the UMAP (**b**) and summarized in the dot plot, where the color of the dots represents average expression and size represents the percentage of cells within the cluster that express it (**c**). Additional cluster markers are shown in Extended Data Fig. 2a,b. **d**, Selected KEGG pathways and GO biological processes (BP) and molecular functions (MF) that showed significant overrepresentation in the cluster marker genes (color of the dots represents odds ratio from one-sided Fisher exact tests, and size of the dots represents the number of genes enriched in category or cell type; BH-adjusted *P* < 0.1). *P* values for individual marker genes and pathways are provided in Source Data Fig. 1. Source data

**Extended Data Fig. 2:**
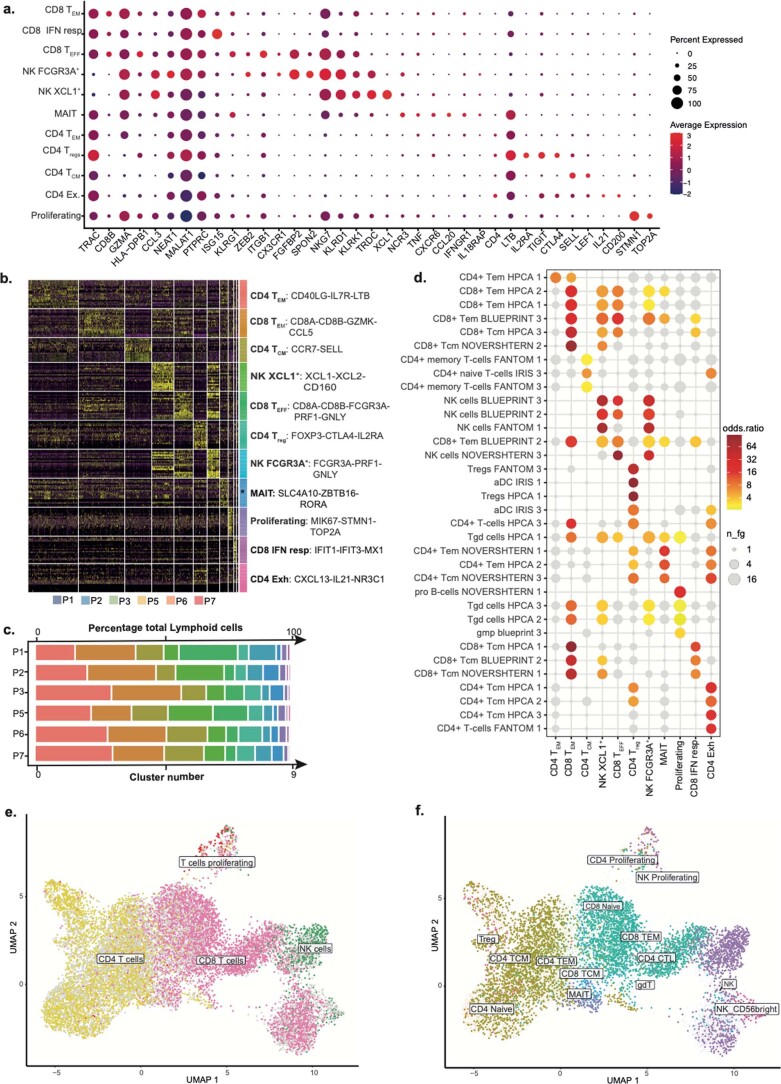
T and NK cell cluster markers and per-patient frequency. Supplementary marker genes for the T and NK populations are shown in the dot plot as (see also Fig. 1c) (**a**). The heatmaps shows the top marker genes for the T and NK cell populations (**b**). The barplots show the frequencies of the T and NK cell clusters in the different patients **(c)**. Gene set over-representation analysis of cluster markers using sets of known cell type marker genes from xCell^17^(**d**). Automatic cell type predictions computed using Azimuth to map the data to the Lung v2 (HLCA) (**e**) and PBMC^18^(**f**) reference datasets.

In total, we found 11 clusters of T and NK cells (Fig. 1a,b). In agreement with previous studies^11^, most T/NK cells present in these human plaques displayed a mature resident phenotype with activation and exhaustion markers such as *CCL5, CD69, NR3C1, PDCD1, KLRG1* and *GZMK*. CD4 T cells formed four clusters: a regulatory T (T_reg_) cell cluster, a central memory T (T_CM_) cell cluster, an effector memory T (T_EM_) cell cluster, and a small cluster displaying high expression of exhaustion markers *CXCL13, NR3C1* and *PDCD1*. CD8 T cell populations included a high *GZMK/IFNG* expressing T_EM_ population, a signature recently associated with inflammaging^15^ and immune activation^16^, and a cytotoxic CD8^+^ effector T (T_eff_) cell cluster. We also noted the presence of a small T cell cluster with expression of interferon (IFN) response genes (including *ISG15*), as well as a cluster with a transcriptional signature compatible with a mucosal-associated invariant T (MAIT) cell subpopulation (*KLRB1, ZBTB16, RORA* and *SLC4A10*). Finally, we found two NK cell populations. The larger population was marked by expression of *XCL1* and *XCL2*, whereas the second *FCGR3A*^*+*^ (CD16^+^) NK cell population shared expression of *PRF1*, *GZMB, GZMH*, *GZMA* and *GNLY* with the CD8 T_eff_ cell cluster (Fig. 1b–d, Extended Data Fig. 2a–c and Source Data Fig. 1). Our manual cluster annotation was consistent with the results of automatic cell-type prediction that was performed by assessing overrepresentation of curated sets of known cell-type markers in the clusters^17^ (Extended Data Fig. 2d) and by mapping cells to reference single-cell datasets with Azimuth^18^ (Extended Data Fig. 2e,f). This analysis confirmed the similarity of the discovered MAIT cell population to blood MAIT cells (Extended Data Fig. 2f), consistent with a recent report that these cells are present in atherosclerotic plaques^19^.

**Fig. 2:**
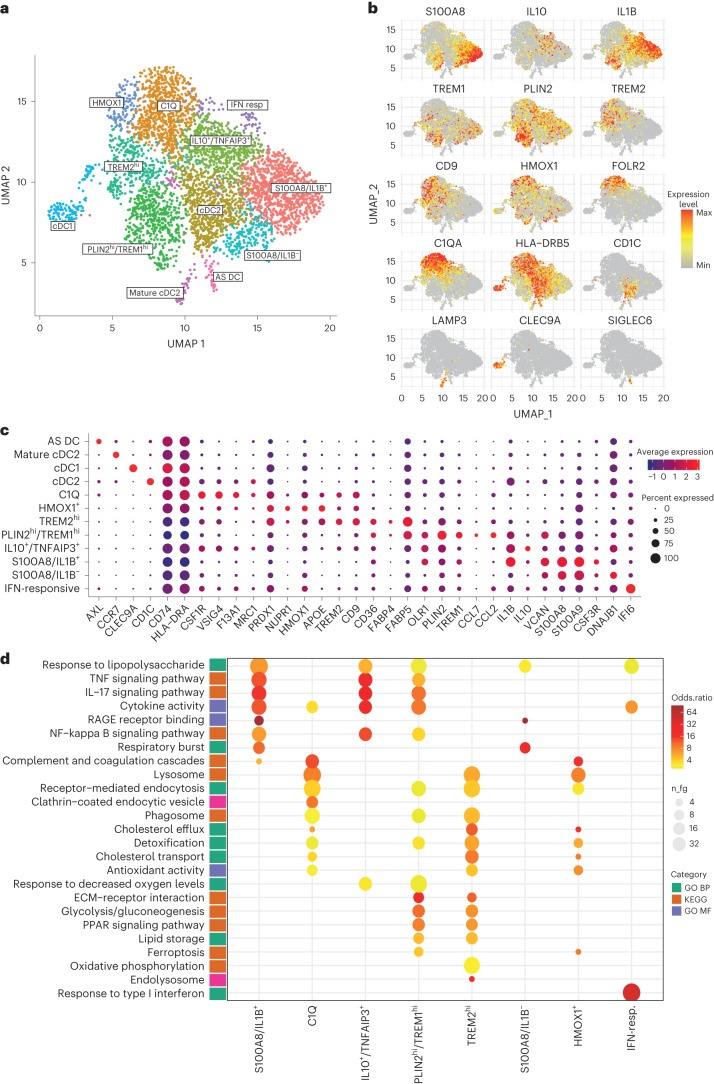
Plaque myeloid cells harbor diverse subsets of macrophages with distinct gene signatures of functional association. **a**–**d**, Cells identified as macrophages or cDCs in the overall analysis (*n* = 4,533 cells; average of *n* = 747 cells per patient) were extracted and analyzed separately. **a**, The UMAP shows the 12 identified myeloid subpopulations. **b**,**c**, The expression of selected automatically discovered cluster marker genes (BH-adjusted *P* < 0.05, two-sided Wilcoxon tests; Extended Data Fig. 3c) and known cell-type marker genes is shown on the UMAP (**b**) and summarized in the dot plot, where the color of the dots represents average expression and size represents the percentage of cells within the cluster that express it (**c**). Additional cluster markers are shown in Extended Data Fig. 3a–c. **d**, Selected KEGG pathways and GO biological processes (BP) and molecular functions (MF) that showed significant overrepresentation in the cluster marker genes (color of the dots represents odds ratio from one-sided Fisher exact tests, and size of the dots represents the number of genes enriched in category or cell type; BH-adjusted *P* < 0.1). *P* values for individual marker genes and pathways are provided in Source Data Fig. 2. Source data

**Extended Data Fig. 3:**
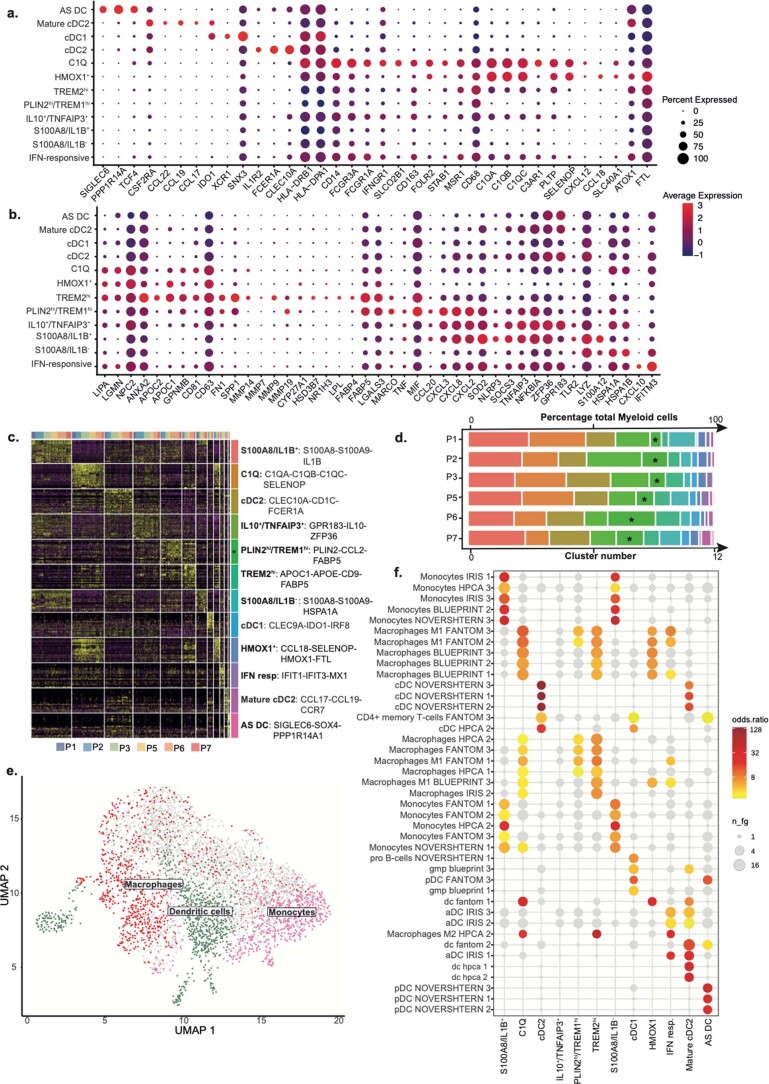
Myeloid cell clusters markers and per-patient frequency. Supplementary marker genes for the myeloid clusters are shown in the dot plots (see also Fig. 2C) **(a, b)**. The heatmaps shows the top marker genes for the myeloid clusters **(c)**. The barplots show the frequencies of the myeloid clusters in the different patients **(D)**. The asterisks in (**c**) and (**d**) mark the position of the PLIN2^hi^/TREM1^hi^ cluster. Automatic cell type predictions performed using Azimuth^18^ to map cells to the Lung v2 (HLCA) reference dataset (**e**). Gene set over-representation analysis of cluster markers using sets of known cell type marker genes from xCell^17^(**f**).

**Extended Data Fig. 4:**
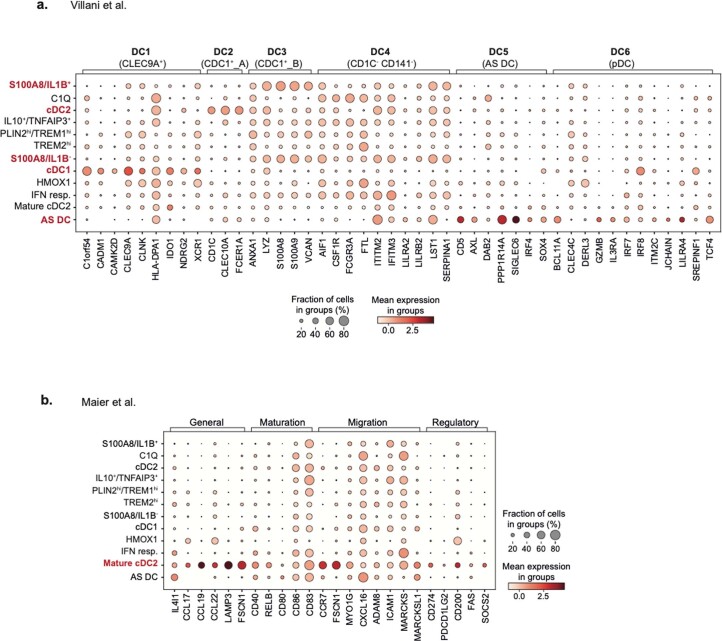
Confirmation of dendritic cell sub-population annotations using known marker genes from the literature. To confirm our annotation of the individual DC subpopulations we examined the expression of known marker genes in our clusters. The dotplot shows the expression of the mReg DC population markers reported in ref. ^20^ (their Fig. 4b) in our myeloid clusters (**a**). The dotplot shows the expression of the DC populations markers reported in ref. ^21^ (their Fig. 1d) in our myeloid clusters (**b**).

**Extended Data Fig. 5:**
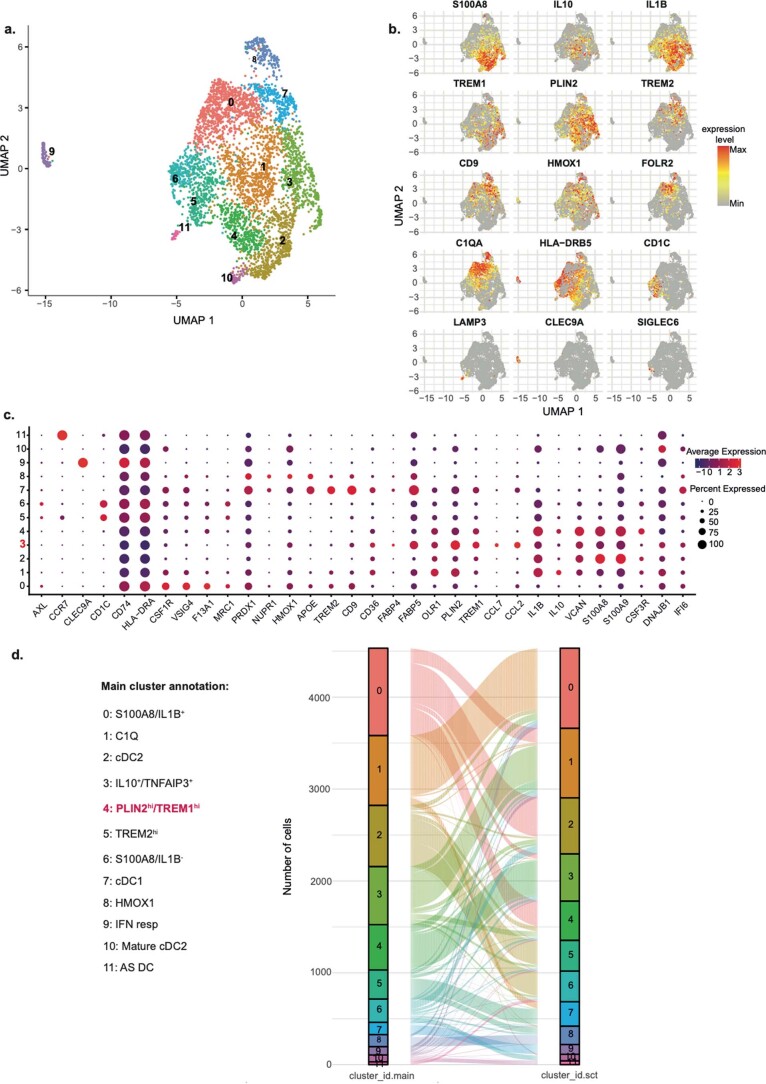
Alternative clustering analysis of myeloid data using a Seurat-based workflow. For this analysis, data normalisation was performed using the sctransform algorithm^23^ and integration was performed using Seurat^24^ as described in the methods. The UMAP shows the identified clusters (Louvain clustering algorithm) (**a**). The expression of selected markers of each cluster are shown on UMAP (**b)**. The dotplot shows the expression of the marker genes from Fig. 2c in the Seurat clusters (cluster 3 comprises of PLIN2^hi^/TREM1^hi^ macrophages) (**c**). The Alluvial plot shows the mapping between the clusters from Fig. 2 (cluster_id.main) and the clusters identified using the Seurat based workflow (Cluster_id.sct) (nearly all of the cells in the PLIN2^hi^/TREM1^hi^ cluster from Fig. 2 map to cluster 3 from the Seurat-based workflow) (**d**).

The in-depth analysis of MNPs (*n* = 4,533 cells) identified four conventional dendritic cell (cDC) clusters and eight macrophage populations (Fig. 2a–d, Extended Data Fig. 3a–f and Source Data Fig. 2). As expected, the cDC clusters included a cDC2 (*CD1C, CLEC10A* and *FCER1A*) subset and a cDC1 (*CLEC9A, IRF8* and *SNX3*) cluster. In addition, we found two subpopulations not yet reported in single-cell studies of human plaques. The first subpopulation was a mature cDC2 cluster that expressed the immune checkpoint genes (*CD40, CD200, TNFRSF4* (encoding OX40) and *CD274* (encoding PDL-1), as well as *LAMP3, MARCKSL1* and *IDO1*) that are compatible with the newly characterized mature DCs enriched in immunoregulatory molecules (mregDCs) in cancer^20^ (Extended Data Fig. 4a). The second subpopulation consisted of recently described *AXL* and *SIGLEC6* (AS)-expressing DCs^21^ that featured genes related to both cDC2 and plasmacytoid DCs (pDCs) (as shown in Extended Data Fig. 4b), and currently has no known role in disease. The remaining eight MNP subsets included a C1Q cluster with efferocytic function, an HMOX1^+^ cluster, two LAM clusters (the previously reported TREM2^hi^ subset and an unreported PLIN2^hi^/TREM1^hi^ LAM cluster), a small IFN-responsive cluster, two calgranulin (S100A8/9/12) clusters and an IL-10^+^/TNFAIP3^+^ cluster (Fig. 2a–c, Extended Data Fig. 3a–d and Source Data Fig. 2). The S100A8/IL-1B^−^ MNP cluster was characterized by a lack of inflammatory signature and a stress response signature (*DNAJB1, HSPA1A* and *HSPA1B*) similar to a recently described cluster in the lungs of patients with coronavirus disease 2019 (COVID-19) (ref. ^22^). The S100A8/IL-1B^+^ MNP cluster was characterized by the expression of genes in the inflammasome pathway: *IL1B*, *NLRP3*; pro-inflammatory cytokines or chemokines *TNF, CCL3, CCL4* and *CCL20*, pro-inflammatory transcription factors *CEBPB* and *NFKB1* and receptor *TLR2* and genes related to senescence and apoptosis (*CDKN1*, *PTGER2, PTGS2* and *MCL1)* (Fig. 2b and Extended Data Fig. 3a,b). Although it shared a high expression of *IL1B* with S100A8/IL1B^+^ MNP, the IL-10^+^/TNFAIP3^+^ MNP cluster was characterized by the additional expression of the anti-inflammatory cytokine *IL10*, the nuclear factor κB (NF-κB) inhibitor *TNFAIP3* and the anti-inflammatory receptor *GPR183*. A similar cluster was identified in murine atherosclerosis^10^. Overall, our annotation of the myeloid phenotypes was consistent with predictions from automatic cell identification algorithms (Extended Data Fig. 3e,f). However, these algorithms suggested that the *S100A8* clusters may have been comprised of monocytes. Finally, we reanalyzed the MNP data using an alternative Seurat-based workflow^23,24^. This analysis confirmed that identification of the PLIN2^hi^/TREM1^hi^ LAM subpopulation was robust to the choice of normalization and integration algorithm (Extended Data Fig. 5a–d).

### *PLIN2*^hi^/*TREM1*^hi^ inflammatory LAMs in human carotid plaques

Next, we sought to characterize the identified macrophage populations in more detail. The C1Q cluster is defined by high expression of the complement family (*C1QA, C1QB* and *C1QC*). The C1q complement is known to enhance phagocytosis and efferocytosis^25^, have anti-inflammatory function through inhibition of TLR signaling^26^ and anti-atherogenic action through binding of intravenous immunoglobulin (IVIg)^27^. The HMOX1^+^ cluster expressed genes involved in heme degradation (*HMOX1*), iron processing and export (*FTL*, *SLC40A1* and *NUPR1*), and antioxidative function (*SELENOP* and *PRDX1*), as well as lysosomal proteases (*CTSB* and *CTSD*), lysosomal genes (*LAMP2, LGMN, LIPA* and *GPNMB*) and genes involved in lipoprotein metabolism (*APOC2, APOE, LRP1* and *NPC2*) (Fig. 2c,d and Extended Data Fig. 3a–c), sharing features with previously described populations in murine plaques^28,29^.

Next, we investigated the phenotype of the PLIN2^hi^/TREM1^hi^ and TREM2^hi^ LAM clusters. These populations shared a lipid-associated transcriptional signature that included fatty acid-binding proteins (*FABP4* and *FABP5*) and lipid scavenger receptors (*CD36* and *MARCO*). As previously described^8,11^, the transcriptional profile of the TREM2^hi^ LAM cluster is consistent with the expression of *NR1H3—*the gene encoding the transcription factor LXRa and a signature of lipid uptake, lysosomal metabolism, antioxidative functions, matrix remodeling, cholesterol metabolism and efflux, with a notable lack of inflammatory genes (Fig. 2c,d and Extended Data Fig. 3a–c). The PLIN2^hi^/TREM1^hi^ LAM cluster was previously unidentified and is characterized by the unique combination of the highest expression of *PLIN2*, which encodes perilipin 2, a protein that coats intracellular lipid droplets and a lipid storage marker^30^, and the innate immune receptor *TREM1*. The PLIN2^hi^/TREM1^hi^ cluster lacked expression of genes involved in lysosomal degradation and cholesterol efflux, but it expressed the inflammatory genes *TREM1, TNF, CEBPB* and *IL1B*. The expression of genes involved in apoptosis, antiproliferation and survival such as *G0S2, BTG1, BCL2A1, IER3*, *BNIP3L* and *MCL1* suggested that cells in this cluster may have been undergoing apoptosis. Among all MNPs, the PLIN2^hi^/TREM1^hi^ LAMs expressed a unique chemokine signature with transcripts for CCR2 ligand *CCL2* and *CCL7*, as well as *CCL20, CXCL1, CXCL2, CXCL3* and *CXCL8*, which was shared with inflammatory MNP clusters (Fig. 2c,d and Extended Data Fig. 3a–c).

**Fig. 3:**
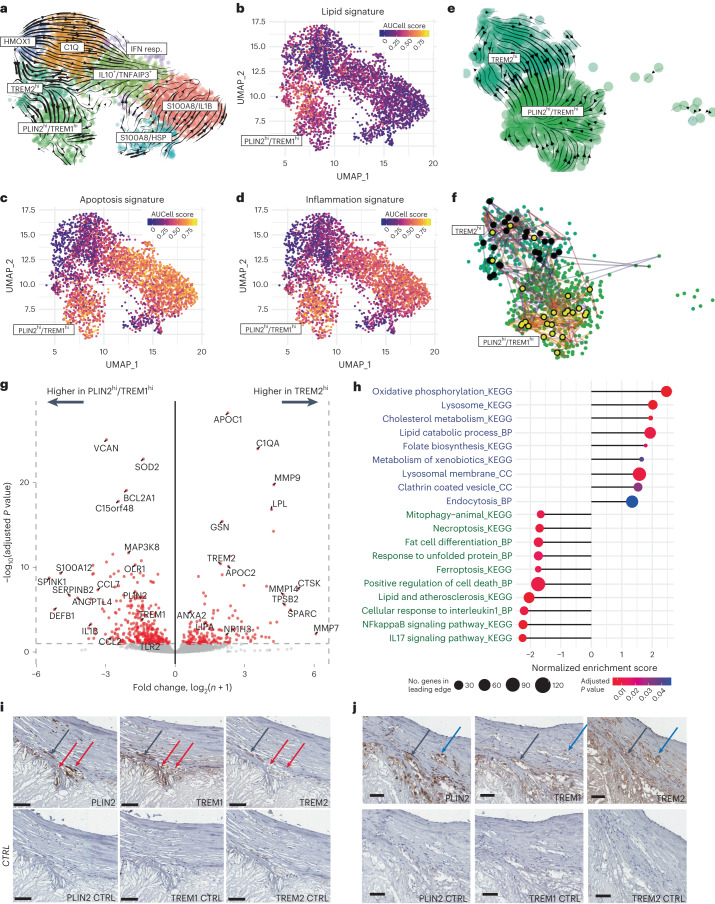
Trajectory analysis of plaque macrophage populations. **a**–**j**, The plaque macrophages (*n* = 3,628 cells; see Fig. 2) were extracted, and RNA-velocity analysis^70^ was performed on the relationship between the eight macrophage clusters. **a**, The arrows on the UMAP indicate the directions of the predicted future transcriptional states of the cells. **b**–**d**, Per-cell scores for lipid metabolism (**b**), apoptosis (**c**) and inflammation (**d**) were computed with AUCell^82^ using custom gene lists (see Methods, Supplementary Table 1) and visualized on the UMAP. **e**,**f**, Targeted RNA-velocity analysis (**e**) and CytoTRACE random walk analysis^85^ (see also Extended Data Fig. 8e) (**f**) of the TREM2^hi^ and PLIN2^hi^/TREM1^hi^ populations. **g**, The volcano plot shows genes differentially expressed between the TREM2^hi^ and PLIN2^hi^/TREM1^hi^ populations. Significantly differentially expressed genes are in red (DESeq2 patient-level pseudobulk analysis, paired Wald tests, BH-adjusted *P* < 0.1). Additional pairwise analysis between PLIN2^hi^/TREM1^hi^ and all other macrophage clusters is shown in Extended Data Fig. 8. **h**, Selected GO and KEGG pathways associated with gene expression differences between the TREM2^hi^ and PLIN2^hi^/TREM1^hi^ populations (FGSEA analysis, genes ranked by the DESeq2 test statistic, BH-adjusted *P* values < 0.05). **i**,**j**, PLIN2^+^/TREM1^+^ (red arrows), PLIN2^+/^TREM2^+^ (blue arrows) and TREM1^+^/TREM2^+^/PLIN2^+^ (black arrows) plaque areas shown by immunostaining on human carotid plaque specimens. Scale bars, 100 μm (staining was performed on *n* = 5 plaques). Source data

**Extended Data Fig. 6:**
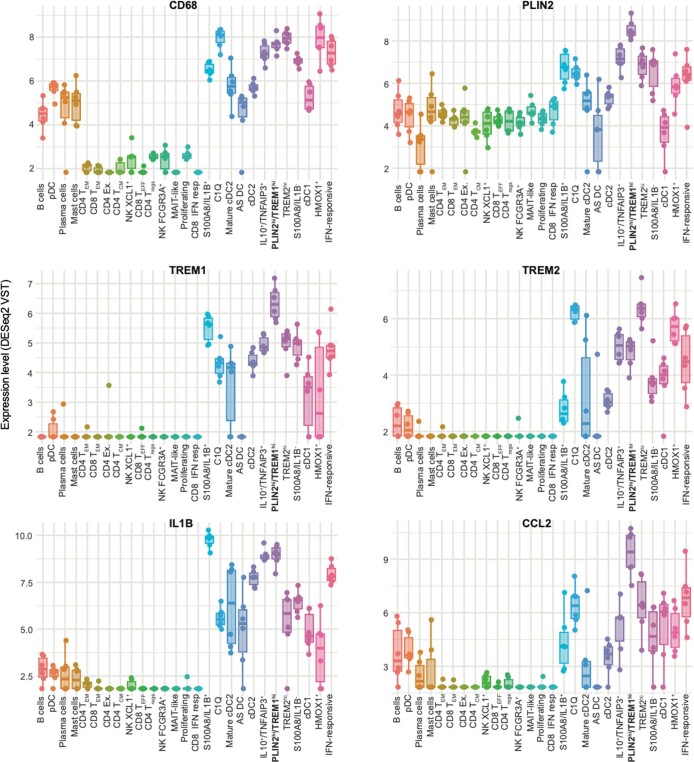
Expression of PLIN2^hi^/TREM1^hi^ signature genes in all identified immune cell populations. The boxplots show the expression of *CD68*, *PLIN2*, *TREM1*, *TREM2*, *IL1B* and *CCL2* in all the immune cell clusters identified. The clusters correspond to those shown in Suppl. Figure 1 (for the B, Plasma, Mast and pDC populations), Fig. 2 (for the T/NK cell clusters) and Fig. 3 (for the myeloid clusters). Normalised per-patient pseudobulk expression values were computed with DESeq2 (Variance Stabilising Transformation). The lower and upper bounds of the boxes mark the first and third quartiles. Line within boxes represent median values. Whiskers extend to the smallest and largest values no further than 1.5 * inter-quartile range from the bounds of the box. Outlying points beyond the whiskers are plotted individually.

**Extended Data Fig. 7:**
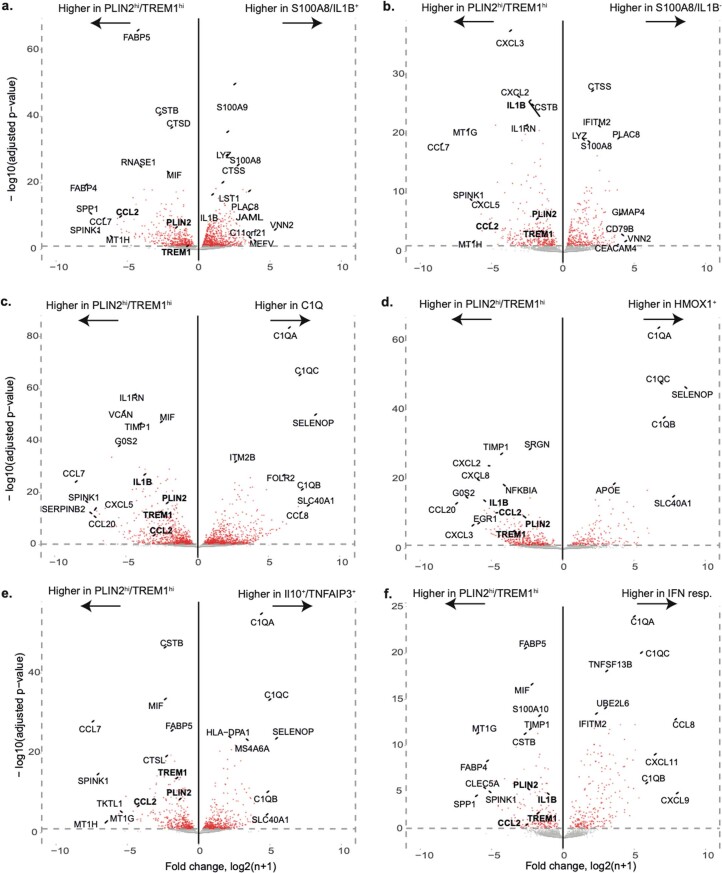
Pairwise differential expression analyses between the PLIN^hi^/TREM1^hi^ macrophages and all the other macrophage populations. The volcano plot shows genes differentially expressed between the PLIN2^hi^/TREM1^hi^ and S100A8/IL1B^+^(**a**), S100A8/IL1B^−^(**b**), C1Q(**c**), HMOX1^+^(**d**), IL10^+^/TNFAIP3^+^(**e**) and IFNresp (**f**) macrophage populations. Significantly differentially expressed genes colored red (DESeq2 patient-level pseudobulk analysis, BH adjusted p < 0.1, list of DEG genes provided in Source Data Fig. 3). *PLIN2*, *TREM1* and *CCL2* (in bold) are consistently significantly more expressed in PLIN2^hi^/TREM1^hi^ cells compared to all other clusters.

Weaker expression of *PLIN2* was observed in foamy TREM2^hi^ and S100A8/IL-1B^+^ MNPs (Fig. 2b,c), but we noted that its expression, along with that of *CCL2* and *TREM1*, was highest in the PLIN2^hi^/TREM1^hi^ subset compared with all other myeloid and nonmyeloid subsets (Extended Data Fig. 6). To further characterize these cells, we performed pseudobulk-level differential expression analyses to compare the PLIN2^hi^/TREM1^hi^ cluster with all of the other myeloid subpopulations. These analyses confirmed that the expression of *PLIN2, TREM1* and *CCL2* was significantly higher in the PLIN2^hi^/TREM1^hi^ subset than in any of the other myeloid clusters across the patient samples (Extended Data Fig. 7a–e and Source Data Fig. 3).

### PLIN2^hi^/TREM1^hi^ LAMs in community scRNA-seq datasets

**Extended Data Fig. 8:**
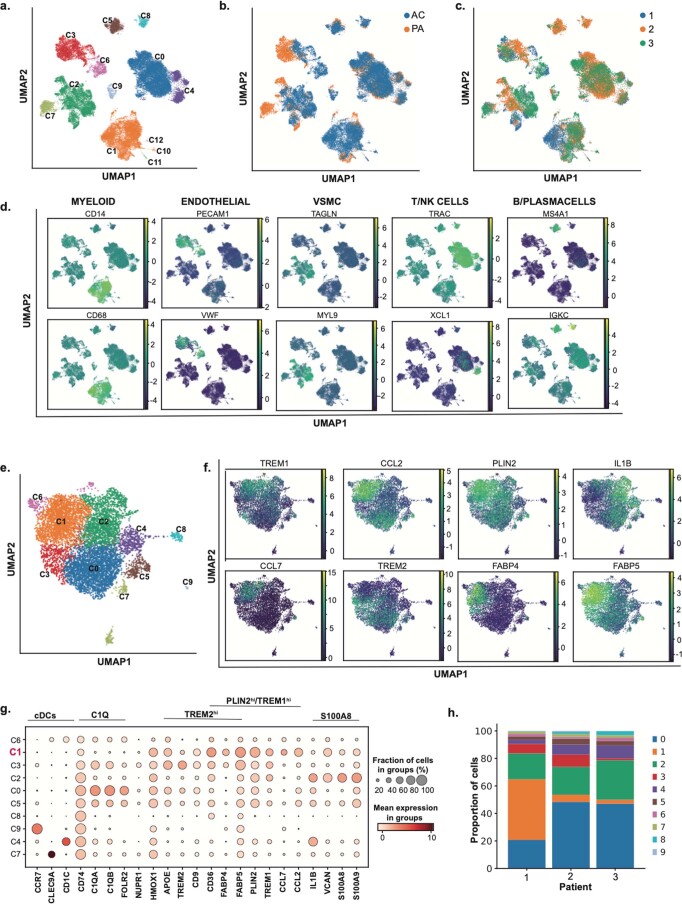
Re-analysis of published human carotid atherosclerosis scRNA seq data (GSE159677)^32^. Unsupervised Leiden clustering of the dataset identified n = 13 distinct clusters (**a**) distributed in both atherosclerotic core (AC) and proximal adjacent (PA) portions of carotid artery tissue (**b**) and in all 3 studied patients (**c**). Identification of the main immune and non-immune clusters was performed using markers used in Alsaigh et al. Figure 1d (**d**). Cells identified as myeloid were extracted and analysed separately. The UMAP shows the 10 identified myeloid sub-populations **(e)**. The expression of selected cluster marker genes is shown on the UMAP **(f)** and summarised in the dot plot (**g**). Distribution of myeloid clusters in the 3 patient samples (**h**).

Next, we sought to establish whether PLIN2^hi^/TREM1^hi^ macrophages are a reproducible feature of human atherosclerosis. We reanalyzed the macrophage populations from two available community scRNA-seq datasets. The researchers in ref. ^31^ performed single-cell analysis of coronary atherosclerotic plaques from eight patients. We found that the majority of the macrophages from this study had a phenotype similar to our C1Q cluster, likely due to inclusion of the coronary adventitia (Supplementary Fig. 2a–c). The researchers in ref. ^32^ performed a single-cell analysis of three human carotid atherosclerosis specimens. We reanalyzed the myeloid cells separately and identified ten clusters of myeloid cells (Extended Data Fig. 8a–h). These included two LAM populations expressing *CD36, FABP4* and *FABP5* that were marked by high expression of *TREM1*, *PLIN2* and *CCL2* (C1) or by high expression of *TREM2* (C3), respectively (Extended Data Fig. 8g).

Next, we investigated whether an equivalent of the human PLIN2^hi^/TREM1^hi^ LAM population exists in mouse atherosclerosis models by performing an integrated analysis of six existing murine myeloid datasets^10,33–37^. In these mouse datasets, we found that *Trem1* was exclusively expressed by monocytes (Supplementary Fig. 3a–c). A gene signature comprised of 1:1 gene orthologs of human TREM2^hi^ LAM marker genes was found to be highly expressed in murine Trem2^hi^ macrophages, while a gene signature similarly derived from human PLIN2^hi^/TREM1^hi^ LAMs was predominately expressed in murine monocytes (Supplementary Fig. 3d–g and Supplementary Table 1). These observations were confirmed by cross-species transfer of the cluster labels using the scANVI algorithm^38^ (Supplementary Fig. 3h). The apparent absence of an obvious murine macrophage phenotype similar to that of human PLIN2^hi^/TREM1^hi^ LAMs (Supplementary Fig. 3h) suggests that there may be a divergent division of labor between monocytes and macrophages or that the duration of current mouse models may not be sufficient to reproduce this feature of human disease.

### Trajectory analysis links TREM2^hi^ to PLIN2^hi^/TREM1^hi^ LAMs

**Extended Data Fig. 9:**
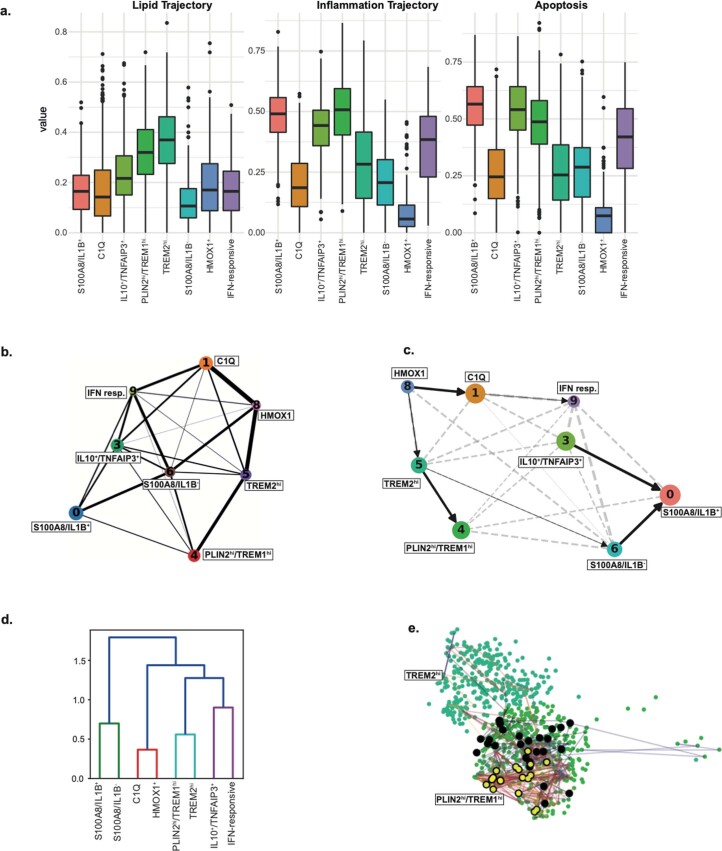
LAM genset scores and macrophage trajectory analysis. Boxplots for the distributions of the lipid, inflammation and apoptosis scores for each of the MNP clusters (n = 3628 cells in total) (**a**). The connectivity between MNP clusters was assessed by partition-based graph abstraction (PAGA) analysis (nodes size and edge width proportional to cluster cell number and degree of connectivity respectively)^39^ **(b)** and PAGA on velocity (**c**). The dendrogram shows the distance between the expression profiles of the clusters (**d**). CytoTRACE random walk analysis where PLIN2^hi^/TREM1^hi^ was assigned as start cluster **(e)**. For the boxplots, lower and upper bounds of boxes mark first and third quartiles. Lines within boxes represent median values. Whiskers extend to the smallest and largest values no further than 1.5 * inter-quartile range from the box. Outlying points beyond whiskers are plotted individually.

To further explore the phenotypes of our human plaque macrophages, we computed per-cell lipid handling, inflammation and apoptosis scores using sets of genes related to each process (see Methods, Supplementary Table 2). This analysis confirmed that the PLIN2^hi^/TREM1^hi^ subset was the only one in the plaque to simultaneously display signatures of lipid handling, inflammation and apoptosis, suggesting that they represented a terminal inflammatory LAM state (Fig. 3a–d and Extended Data Fig. 9a).

Next, we applied partition-based graph abstraction (PAGA) analysis^39^ to assess the similarity between the MNP clusters. The highest degree of connectivity was observed between the C1Q, HMOX1, TREM2^hi^ and PLIN2^hi^/TREM1^hi^ populations. The two calgranulin-expressing and IL10^+^/TNFAIP3^+^ populations were adjacent in the graph but showed weaker connectivity (Extended Data Fig. 9b,c).

RNA-velocity analyses on uniform manifold approximation and projection (UMAP) and PAGA graphs supported the presence of three putative differentiation trajectories within the MNP clusters (Fig. 3a and Extended Data Fig. 9d): (1) from HMOX1^+^ to C1Q, (2) from S100A8/IL1B^−^ to the overtly pro-inflammatory S100A8/IL1B^+^ population, and (3) from TREM2^hi^ to inflammatory PLIN2^hi^/TREM1^hi^ LAMs. The predicted connection between TREM2^hi^ and PLIN2^hi^/TREM1^hi^ was of particular interest because it linked the two putative LAM states. We therefore applied a second, RNA-velocity-independent approach to investigate the possibility that LAMs might transition between these two phenotypes. Consistent with the RNA-velocity results, random walk analysis (CellRank, CytoTRACE kernel) also predicted that TREM2^hi^ could differentiate into inflammatory PLIN2^hi^/TREM1^hi^ LAMs (Fig. 3e,f) but did not provide support for differentiation in the opposite direction (Extended Data Fig. 9e).

Then, we performed a targeted investigation of the differences between the two LAM states. As expected, the PLIN2^hi^/TREM1^hi^ LAMs showed significantly higher expression of *PLIN2* along with higher expression of the apoptosis-related gene *SPINK1*, the pro-inflammatory cytokine *IL1B*, chemokine *CCL2*, *TLR2* and *VCAN* (which encodes the TLR2 ligand versican^40^). PLIN2^hi^/TREM1^hi^ displayed relative pathway enrichments for genes associated with apoptosis and inflammation. In contrast, TREM2^hi^ showed higher expression of the matrix metalloproteinases *MMP7, MMP9* and *MMP14*, as well as *LPL* and *C1QA*, along with pathway enrichments for oxidative phosphorylation and cholesterol metabolism (Fig. 3g,h and Source Data Fig. 3).

Finally, we investigated the spatial niches occupied by PLIN2^hi^/TREM1^hi^ and TREM2^hi^ LAMs in situ in human plaques. Immunohistochemistry showed that the two LAM subsets were found in different locations: TREM2^+^/TREM1^−^ LAMs predominantly were superficially located and adjacent to the fibrous cap, whereas TREM1^+^/TREM2^−^ LAMs were located deeper in the lipid core, close to lipid clefts. Cells expressing both TREM2 and TREM1 were also identifiable in the proximity of both subsets, lending support to the concept of a transition within LAMs (Fig. 3i,j).

### TLR signaling mediates the transition between the two LAMs

**Fig. 4:**
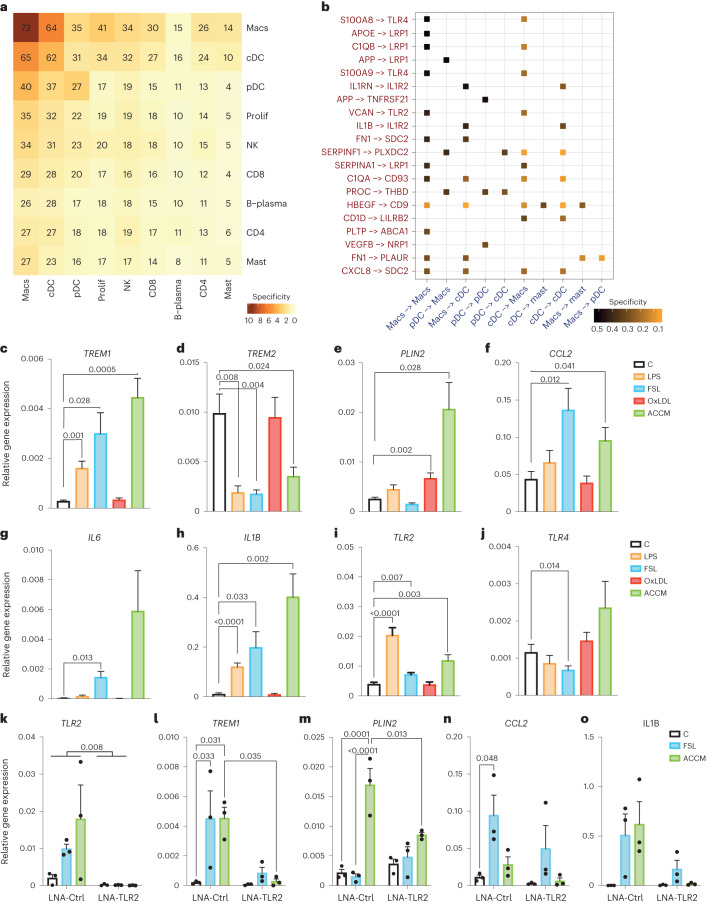
Cell–cell interaction analysis suggests a central role for macrophages in the immune cell communication network of the human atherosclerotic plaque. **a**–**o**, Cell–cell interactions between major immune cell types were investigated using NATMI^41^ to determine the number of ligand–receptor pairs that connected each pair of cell types. **a**, The overall cell-connectivity-summary network is summarized in the heat map (cells expressing ligands are shown in rows, and cells expressing receptors are shown in columns). The number of significant ligand–receptor pairs is indicated for each interaction. The heat map is colored according to the NATMI specificity score (product of ligand specificity × receptor specificity). **b**, Selected examples of predicted ligand–receptor interactions (*y* axis; ordered by specificity) between the given source and target cell populations (*x* axis) are shown in a heat map. **c**–**j**, Gene expression of *TREM1* (**c**), *TREM2* (**d**), *PLIN2* (**e**), *CCL2* (**f**), *IL6* (**g**), *IL1B* (**h**), *TLR2* (**i**) and *TLR4* (**j**) was measured in hMDMs treated with TLR4 ligand (LPS, 1 ng ml^−1^), TLR2 ligand (FSL-1, 100 ng ml^−1^), oxLDL (25 μg ml^−1^) or ACCM for 24 h. Data are reported as relative gene expression compared with housekeeping gene (*n* = 17 biologically independent donors from seven independent experiments for all genes, with the exception of *IL6* where *n* = 13 biologically independent donors were analyzed from five independent experiments; values reported as mean ± s.e.m., one-way analysis of variance (ANOVA), mixed-effect analysis). Blocking TLR2 in hMDMs abrogated the effect of ACCM. hMDMs were treated with LNA-ASOs targeting TLR2 (Methods) for 3 days prior to a 24-h treatment with TLR2 ligand (FSL-1, 100 ng ml^−1^) or ACCM. **k**–**o**, Gene expression of *TLR2* (**k**), *TREM1* (**l**), *PLIN2* (**m**), *CCL2* (**n**) and *IL1B* (**o**) was measured. Data are reported as relative gene expression compared with housekeeping gene (*n* = 3 biologically independent donors from two independent experiments; values reported as mean ± s.e.m., two-way ANOVA). Source data

To identify candidate drivers of the TREM2^hi^ to PLIN2^hi^/TREM1^hi^ LAM transition, we searched for potential ligand–receptor interactions between the major cell types present in the plaque using the Network Analysis Toolkit for Multicellular Interactions (NATMI)^41^. The highest number of potential interactions and ligand–receptor pair specificities were found within the MNP compartment (Fig. 4a). They included ligand interactions with TLR2 (*VCAN_TLR2)* and TLR4 (*S100A8/9_TLR4)* (Fig. 4b), as well as interactions with the low-density lipoprotein receptor-related protein (*LRP1*) that are associated with pathways involved in pro-inflammatory signaling and lipid handling, respectively.

**Extended Data Fig. 10:**
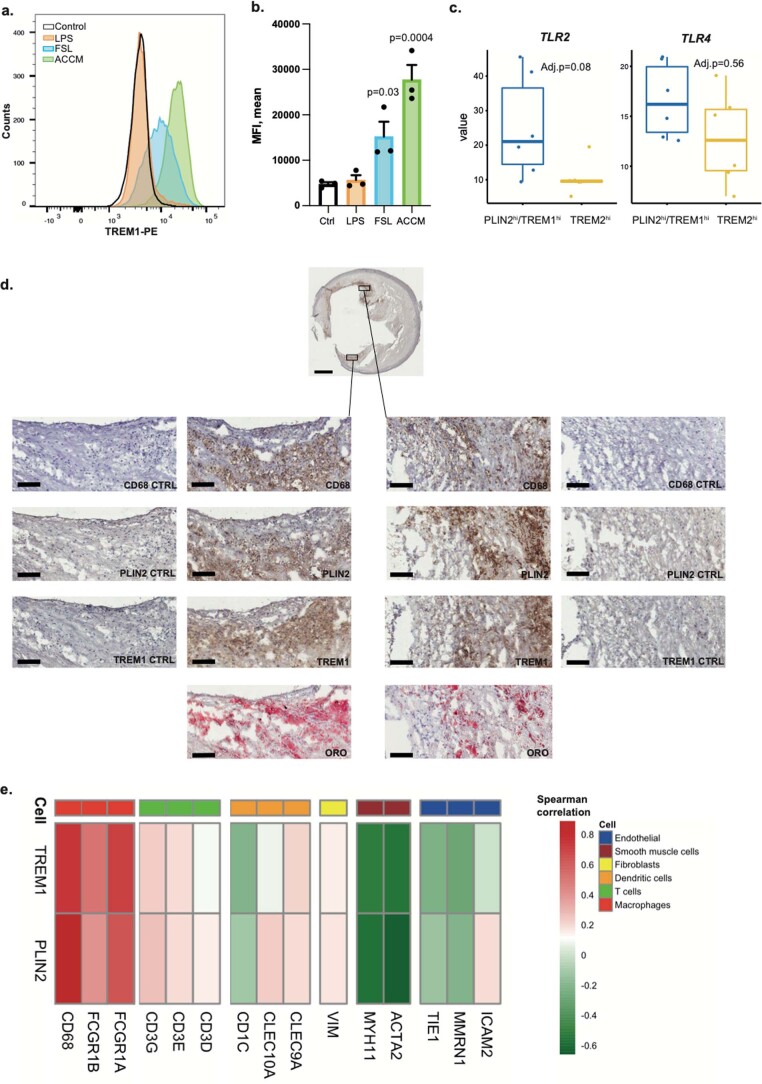
TREM1 protein expression in all treatment groups was measured using flow cytometry (representative of n = 3 biologically independent represented (**a)**). Quantification of mean MFI from n = 3 biologically independent samples (values reported as mean ± SEM, One way ANOVA, Dunnett’s multiple comparison) (**b**). Box plots for TLR2 and TLR4 differential expression in TREM2^hi^ and PLIN^hi^/TREM1^hi^ cluster. TLR2 gene expression is higher in PLIN^hi^/TREM1^hi^ (DESeq2’s Wald test BH adjusted p-value = 0.08) while no significant difference was found for TLR4 (BH adjusted p-value = 0.56) (DESeq2 analysis; two-sided, paired Wald tests) (**c**). The Carotid Plaque Imaging Project (CPIP) biobank samples were stained for CD68, PLIN2 and TREM1 and their respective controls as well as oil red o (ORO) stain. Scale bars 1 mm (far left and far right) and 100um (in the amplified images) (staining for a total of n = 37 plaques was performed)(**d**). The heatmap shows the correlation between TREM1 and PLIN2 expression from CPIP bulk transcriptomic data with markers of different cell types present in plaque. Both TREM1 and PLIN2 only showed high correlations with the macrophage markers. Spearman correlation coefficient was used, n = 60 patients (**e**). For the boxplots, lower and upper bounds of boxes mark first and third quartiles. Lines within boxes represent median values. Whiskers extend to the smallest and largest values no further than 1.5 * inter-quartile range from the box. Outlying points beyond whiskers are plotted individually. Source data

To investigate the ability of candidate factors to promote plaque macrophage phenotypes, we established an ex vivo stimulation assay using human CSF1-dependent monocyte-derived macrophages (hMDMs). To mimic the atherosclerotic plaque soluble milieu, we used atheroma cell conditioned medium (ACCM) as a source of activation^42^. Because the ligand–receptor interactions through TLR2 and TLR4 were highly specific to the predicted macrophage–macrophage interactions (Fig. 4b), we also stimulated the hMDMs with a panel of TLR stimuli, including oxidized low-density lipoprotein (oxLDL), TLR2 ligand FSL-1 and TLR4 ligand LPS. Stimulation with ACCM upregulated *TREM1* and dowregulated *TREM2* gene expression (*TREM1*, *P* = 0.0005; *TREM2*, *P* = 0.024; Fig. 4c,d and Source Data Fig. 4). TREM1 upregulation was also confirmed at the protein level (Extended Data Fig. 10a,b and Source Data Extended Data Fig. 10). Exposure to ACCM also increased the expression of *PLIN2* (*P* = 0.03), *CCL2* (*P* = 0.04), *IL1B* (*P* = 0.002) and *TLR2* (*P* = 0.003) (Fig. 4e–i). This demonstrates that soluble mediators derived from atherosclerotic plaque-resident cell populations can activate inflammatory LAM programming.

TLR stimulation of the hMDMs with oxLDL enhanced *PLIN2* expression (*P* = 0.002; Fig. 4e), but did not fully reproduce per se the cluster-specific gene signature of PLIN2^hi^/TREM1^hi^ cells. FSL-1 (TLR2) and LPS (TLR4) stimulation both downregulated expression of *TREM2* (*P* = 0.004 and *P* = 0.008, respectively; Fig. 4d), with TLR2 stimulation significantly upregulating gene and protein expression of *TREM1* (Fig. 4c and Extended Data Fig. 10a,b). FSL-1 (TLR2) also induced PLIN2^hi^/TREM1^hi^ LAM cluster-specific *CCL2* gene expression in the hMDMs (*P* = 0.012; Fig. 4f). These data show that ex vivo TLR signaling can induce an inflammatory LAM phenotype similar to that observed in the atheroma. In support of the concept that the inflammatory LAM state might involve TLR2 signaling, the PLIN2^hi^/TREM1^hi^ plaque LAMs had significantly higher expression of *TLR2* and *VCAN* (Fig. 3g). In contrast, *TLR4* expression did not show significantly higher expression in PLIN2^hi^/TREM1^hi^ LAMs (adjusted *P* = 0.56; Extended Data Fig. 10c), suggesting that TLR2 might be crucial for the inflammatory activation of LAMs.

To functionally implicate TLR2 in the phenotypic switch to inflammatory LAMs, we silenced TLR2 expression in hMDMs using locked nucleic acid antisense oligonucleotides (LNA-ASOs) targeting TLR2 prior to stimulation with the ACCM to model the human atheroma microenvironment. The TLR2 agonist FSL-1 was used as control. LNA-TLR2 achieved efficient knockdown of TLR2 expression in hMDMs (Fig. 4k). Knockdown of TLR2 led to a significant decrease in induction of *TREM1*, *PLIN2*, *IL1B* and *CCL2* gene expression after exposure to ACCM, whereas it prevented decrease in *TREM2* (Fig. 4l–o and Source Data Fig. 4). FSL-1-dependent gene induction was also prevented (Fig. 4l–o), albeit FSL-1 and ACCM differed in their ability to modulate *PLIN2* expression (Fig. 4c–j), suggesting differences in the biological action of exogenous and endogenous TLR2 agonists.

### PLIN2^hi^/TREM1^hi^ signature correlates with vascular events

**Fig. 5:**
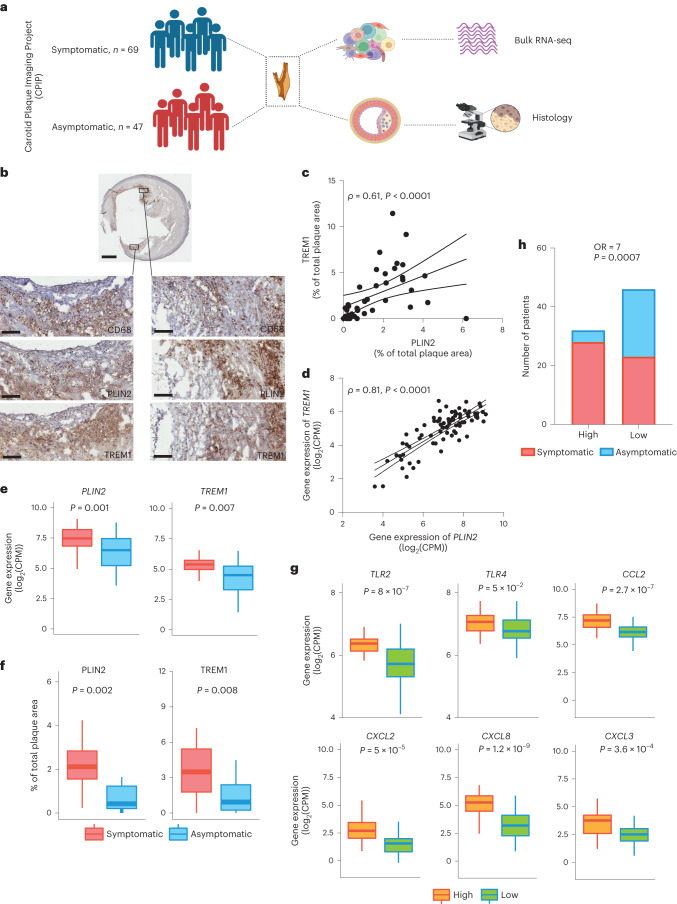
Symptomatic plaques have more cells associated with the inflammatory TREM1 LAM signature. **a**, The CPIP biobank samples were used as validation cohort following the illustrated diagram (diagram created by BioRender). **b**, Plaque areas positive for CD68, PLIN2, TREM1 and Oil Red O were shown to stain the same areas (marked by rectangles) by immunostaining on human carotid plaque specimens. Scale bars, 1 mm (whole carotid section image) and 100 μm (in the magnified images; representative image from *n* = 37 stained plaques). An additional staining example with the corresponding antibody controls is shown in Extended Data Fig. 10a. **c**, The plaque area that stained positive for TREM1 correlated positively with the PLIN2 plaque area (Spearman test was used for the correlation analysis; *n* = 37). **d**, *TREM1* and *PLIN2* gene expression, assessed by bulk RNA-seq in carotid plaque samples collected from the CPIP biobank, were strongly correlated (*n* = 78; Spearman rank correlation test). **e**,**f**, Plaque areas stained positive (% of total plaque area) for *PLIN2* and *TREM1* (*n* = 19 asymptomatic and *n* = 18 symptomatic; Mann–Whitney *U*-test was used for group comparisons; **e**), as well as gene expression levels of *PLIN2* and *TREM1* (*n* = 51 symptomatic and *n* = 27 asymptomatic; Student’s *t*-test was used for group comparisons; **f**), were significantly higher in symptomatic plaques than in asymptomatic plaques. **g**, Human carotid plaque gene expression levels of *TLR2*, *TLR4*, *CCL2*, *CXCL2*, *CXCL3* and *CXCL8* comparing TREM1/PLIN2 high-expressing and TREM1/PLIN2 low-expressing plaques from the CPIP cohort (see Methods). Data are presented as log_2_(CPM) compared between the two groups using two-sided Student’s *t*-test. **h**, Distribution of symptomatic and asymptomatic patients in TREM1/PLIN2 high-expressing versus TREM1/PLIN2 low-expressing plaques from the CPIP cohort (*n* = 32 high and *n* = 46 low, OR = 7, *P* = 6.6 × 10^−4^, Fisher exact test). Boxes indicate interquartile range (IQR; 25th and 75th percentiles); center line indicates median (50th percentile); whiskers indicate minimum (within lower quartile − 1.5 × IQR) to maximum (within upper quartile + 1.5 × IQR).

To understand the clinical relevance of PLIN2^hi^/TREM1^hi^ inflammatory LAMs, we used the CPIP biobank samples as a validation cohort (Fig. 5a and Supplementary Table 3). We performed immunohistochemistry on 37 carotid plaque sections (*n* = 19 asymptomatic and *n* = 18 symptomatic). We found a colocalization of TREM1 and PLIN2 in plaque areas positive for CD68 and Oil Red O (neutral lipids) (Fig. 5b and Extended Data Fig. 10d). Quantification of the immune-positive areas demonstrated a positive and significant correlation between TREM1 and PLIN2 staining (*ρ* = 0.61, *P* < 0.0001, Spearman test; Fig. 5c).

Targeted analysis of bulk transcriptomic profiles from 78 carotid endarterectomies (*n* = 51 symptomatic and *n* = 27 asymptomatic) from the CPIP also showed a significant positive correlation between *TREM1* and *PLIN2* gene expression levels (*ρ* = 0.81, *P* < 0.0001, Spearman test; Fig. 5d). In addition, strong positive correlations were found between the expression of both *TREM1* and *PLIN2* and key macrophage marker genes, suggesting that macrophages were the principal source of expression of these genes (Extended Data Fig. 10e). The CPIP cohort data thus demonstrate that coordinated *TREM1* and *PLIN2* expression is also present within undissociated human carotid plaque tissues.

Finally, in the CPIP data and relative to asymptomatic plaques, plaques from symptomatic patients show higher gene expression, as well as greater plaque area stained positive for TREM1 and PLIN2 (Fig. 5e,f). Plaques with high *PLIN2/TREM1* expression (determined using a tertile scoring system described in Methods) displayed higher gene expression levels of *TLR2* (*P* = 8 × 10^−7^) along with higher expression of chemokines found to be transcribed by this population in the single-cell analysis (*CCL2*, *P* = 2.7 × 10^−7^; *CXCL2*, *P* = 5.0 × 10^−5^; *CXCL3*, *P* = 3.6 × 10^−4^; and *CXCL8*, *P* = 1.2 × 10^−9^) compared with plaques with low *PLIN2*/*TREM1* expression (Fig. 5g), reproducing the unique chemotactic profile features of these cells. Plaques with high PLIN2^hi^/TREM1^hi^ signature gene profiles were also significantly more likely to belong to patients who recently experienced stroke or transient ischemic attack (odds ratio (OR) = 7, *P* = 6.6 × 10^−4^, Fisher exact test; Fig. 5h), further underscoring the association of this macrophage transcriptional signature with symptomatic atherosclerosis. This validation study in the CPIP cohort supports our identification of the PLIN2^hi^/TREM1^hi^ LAMs by scRNA-seq and indicates that PLIN2^hi^/TREM1^hi^ macrophages are associated with carotid plaque complications leading to cerebrovascular events.

## Discussion

Atherosclerotic plaque composition is a key determinant of coronary and cerebrovascular syndromes in humans. A deep understanding of the cellular transcriptional states underpinning human atherosclerotic plaque composition allows for the identification of targetable cellular drivers. Our study of the human atheroma provides a high-resolution atlas of the immune cell landscape of this tissue. In our myeloid atlas, known subsets were identified, including TREM2^hi^, C1Q and calgranulin-expressing MNPs^11,43–45^. We resolved expected subsets not yet reported in single-cell biology studies of human atherosclerosis, that is, HMOX1^+^ macrophages sharing features with previously described populations in murine plaques^28,29,46^, a mature cDC2 cluster previously identified in cancer^20^ expressing a plethora of immune checkpoints with a role in atherogenesis^47,48^, *AXL* and *SIGLEC6* (AS)-expressing DCs^21^, and IL-10^+^/TNFAIP3^+^ MNPs expressing both extracellular (IL-10) and intracellular (A20) means to antagonize NF-κB-driven inflammation and atherogenesis^45,47^. Finally, we identified a previously unknown subset of TLR-dependent PLIN2^hi^/TREM1^hi^ inflammatory LAMs, whose signature is enriched in carotid plaques of patients who experienced a cerebrovascular event.

To date, single-cell studies have suggested a dichotomy between lipid-associated and inflammatory macrophage states in atherosclerosis. LAMs identified in several human diseases^6–8,49,50^ appear to share a common transcriptional program of the TREM2^hi^ macrophages^9^. TREM2 has been repeatedly demonstrated in a number of models as a driver of lipid homeostasis, and its loss is associated with dysregulated cholesterol efflux and consequent cholesterol ester accumulation^8,51,52^. Our dataset reveals that the TREM2^hi^ phenotype is not the only transcriptional state of LAMs. We discovered the transcriptional state of the human inflammatory LAMs in situ, the PLIN2^hi/^TREM1^hi^ macrophages, the sole subset in which transcriptional signatures of lipid loading and inflammation are coupled. Moreover, PLIN2^hi^/TREM1^hi^ LAMs exhibit a unique chemokine signature with transcripts for pro-atherogenic CCR2 ligands *CCL2* and *CCL7* with a nonredundant role in atherogenesis and monocyte recruitment^53^. This transcriptomic signature suggests that PLIN2^hi/^TREM1^hi^ macrophages may help to orchestrate the recruitment of other immune cells within atherosclerotic plaques.

PLIN2 is the main protein associated with lipid droplets in adipocytes and macrophages and is a marker of lipid accumulation^30^. PLIN2 was found to be negatively correlated with cholesterol efflux in macrophages^30^, consistent with the decrease in TREM2, LXR and its downstream ATP-binding cassette (ABC) transporters in the gene signature of PLIN2^hi^/TREM1^hi^. Deficiency of PLIN2 in the murine model of atherosclerosis protects from atherogenesis^30^. In addition, vulnerable plaque in humans was found to have significantly higher expression of perilipin compared with stable plaque^54^. TREM1 acts as a master switch of innate immune responses triggered by pathogens, where it magnifies the pro-inflammatory response by synergizing with classical pattern-recognition receptors (TLR and NOD)^55^. TREM1 promotes lipid accumulation and apoptosis by inhibiting lipid efflux^56,57^ and is linked to a larger necrotic core in murine models of atherogenesis^56,57^, in line with the maladaptive lipid-handling profile of the PLIN2^hi/^TREM1^hi^ macrophages. TREM1 deficiency upregulates TREM2 expression and prevents loss of homeostasis in stroke^51^.

Our study highlights potential similarities and differences between human and murine LAMs in atherosclerosis. Whereas the TREM2^hi^ macrophage transcriptional signature was conserved across both species, this did not appear to be the case for the human inflammatory LAM signature. This is in agreement with existing literature showing that mouse foamy macrophages have a homeostatic signature with little inflammation^10,33,37^. Human disease is a multifactorial and chronic process that spans over several decades^58^, and we cannot exclude the possibility that feeding a high-fat, high-cholesterol diet for longer than in the available datasets might be required to observe an inflammatory evolution of LAMs in mouse aorta.

Macrophage plasticity is thought to be essential for the establishment of context-dependent macrophage subsets^59^. Our trajectory analysis predicts that the TREM2^hi^ macrophage is not a static transcriptional state but that it can transition toward an inflammatory PLIN2^hi^/TREM1^hi^ LAM phenotype. This computational trajectory appeared distinct from the trajectory leading to the calgranulin-expressing MNPs, suggesting that two distinct routes might exist for inflammatory macrophage polarization in the plaque. The predicted transition was phenocopied in vitro by the exposure of human CSF1-dependent macrophages to human atheroma cell conditioned medium, underscoring the importance of the specialized milieu of the plaques for local MNP phenotypes.

Although the biology of TREM1 (refs. ^51,56,57,60^) and TREM2 (refs. ^8,52^) is well established, a mechanism for transition between different LAM states has not been previously elucidated. TLRs are innate immune receptors for microbial patterns and danger signals^61^. TLR2 and TLR4 and downstream signaling adapter MyD88 have pro-atherogenic functions in murine models, and they recognize modified lipoproteins in concert with scavenger receptors contributing to foam cell formation^4,5^. Their role in human atherosclerosis is less well defined. We previously showed that inflammation in human atherosclerosis cell isolates is driven by TLR2, and to a lesser extent TLR4, via the IL-1R/TLR signaling adaptor MyD88 (ref. ^42^). However, the cell responsible for the signaling could not be pinpointed without single-cell biology. TLR2 was differentially expressed in inflammatory LAMs compared with TREM2^hi^ LAMs. Using ligand–receptor interaction analysis and functional studies in vitro, we reveal the key contribution of TLR2 signaling in the LAM transition toward PLIN2^hi/^TREM1^hi^ LAMs, indicating that danger signaling plays a role in LAM switching toward an activated state. We and others^11^ identify VCAN–TLR2 ligand–receptor pairing potentially involved in macrophage–macrophage crosstalk in the human plaque, indicating that potential endogenous ligands may contribute to this phenotypic switching. Further studies are required to identify the precise components of the atherosclerosis milieu that are responsible for TLR signaling.

In further support for the existence of PLIN2^hi/^TREM1^hi^ LAMs, bulk RNA-seq and immunohistochemistry data from the CPIP cohort showed strong correlations, and in the latter, costaining between PLIN2, TREM1 and CD68, excluding the possibility of a tissue digestion artifact. Our immunohistochemistry validation confirms the existence of two distinct LAMs that occupy different topographical niches within the human atherosclerotic plaque, with TREM2^+^/PLIN2^+^ being placed in proximity of the fibrous cap and TREM1^+^/PLIN2^+^ placed deeper in the lipid core, close to lipid clefts. Our study provides reliable markers to readily identify inflammatory LAMs in situ in humans.

The inflammatory LAM signature was significantly associated with a high risk of cerebrovascular events in the CPIP study. Bulk RNA-seq data and immunohistochemistry consistently showed that both gene expression levels and immunopositive areas of TREM1 and PLIN2 were greater in plaques from patients who experienced symptomatic carotid disease. Concomitantly high expression of both TREM1 and PLIN2 in human plaques was associated with higher *CD68*, chemokine and *TLR2* transcripts, in close agreement with the transcriptional features of the PLIN2^hi^/TREM1^hi^ LAMs present in the discovery cohort. Importantly, joint high expression of both TREM1 and PLIN2 in human carotid plaques was associated with a significantly higher incidence of cerebrovascular symptoms compared with plaques with a low expression of both. In summary, we identify PLIN2^hi^/TREM1^hi^ LAMs as an important component of the intraplaque atherogenic inflammatory response that is associated with the occurrence of cerebrovascular events.

In conclusion, our single-cell dataset provides a valuable reference atlas for future studies of human atherosclerotic syndromes. We report the PLIN2^hi^/TREM1^hi^ inflammatory LAM transcriptional state with features of a cellular culprit of disease. We show that TLR signaling has a prominent role in the induction of inflammatory LAMs. Our data reconcile the concept of atherosclerosis as lipid-driven inflammation with the single-cell biology of human atheroma, with substantial relevance to the debate on the inflammatory basis of atherosclerosis and to its translation into clinical practice. These findings underscore the importance of selective targeting of intraplaque lipid-driven inflammatory pathways rather than generic immune-modulation strategies.

## Methods

### Patient population for scRNA-seq data (discovery cohort)

The study was approved by UK National Research Ethics Services (RREC2989 and RREC08/H0706/129). The study population consists of patients who underwent carotid endarterectomy at Oxford University Hospitals National Health Service Trust. Patients gave their written informed consent to have their discarded and anonymized plaque tissue collected as part of the Oxford Peripheral Vascular Disease Study (OxPVD). A total of six patients’ carotid plaques were analyzed for the current study. Patients were defined as symptomatic if they experienced symptoms (stroke, transient ischemic attack or amaurosis fugax) with a carotid plaque with a degree of stenosis greater than 70%. Asymptomatic carotid plaques were associated with no documented clinical symptoms but had indication for carotid endarterectomy due to a high degree of stenosis, according to North American Symptomatic Carotid Endarterectomy Trial (NASCET) or European Carotid Surgery Trial (ECST) criteria^62,63^.

### Carotid plaque processing for single-cell analysis and supernatant collection

Carotid plaques were directly collected at the operation in cold RPMI medium (Gibco, 21875-034) supplemented with 5% fetal bovine serum (FBS; Biosera, FB-1001/500) and processed within 12 h. Cell suspensions from freshly digested tissues were obtained following a previously published protocol^64^. In brief, the tissue was extensively washed in RPMI and finely minced, and tissue fragments were incubated in collagenase type I (400 units ml^−1^; Sigma, C9722), elastase type III (5 units ml^−1^; Worthington, LS006365) and DNase (300 units ml^−1^; Sigma, D5025), with 1 mg ml^−1^ soybean trypsin inhibitor (Sigma, T6522), 2.5 μg ml^−1^ polymixin B (Sigma, P4932) and 2 mM CaCl_2_, in RPMI medium 1640 with 5% FBS in a shaker at 37 °C for 45 min. Cell suspension was filtered through an 80-μm Nylon mesh and washed in media. Collected cells were incubated with live/dead stain at 1:1,000 in PBS at 4 °C for 10 min. Cells were then washed with FACS buffer (PBS with 2% FBS), stained for CD45 antibody (5 μl per 100 μl; BioLegend, 103108) at room temperature (RT, 21 °C) for 30 min, then washed twice with FACS buffer, resuspended in a final FACS buffer volume of 500 μl, filtered through an Easy Strain 100-μm cell strainer and sorted for live CD45^+^ cells using FACSAria III (BD Biosciences) (sorting strategy shown in Supplementary Fig. 4). For the generation of ACCM, human atheroma cells were isolated with the same enzymatic protocol as per scRNA-seq and cultured for 24 h in serum-free medium at 10^6^ cells ml^−1^, followed by centrifugation at 300 × *g* and cryopreservation in aliquots for consistency. Contamination with LPS was excluded by the sensitive limulus amebocyte assay in the ACCM, the enzymatic mixtures and all reagents used for the cell preparation and culture.

### Generation of droplet-based scRNA-seq data

Sorted live CD45^+^ cells were resuspended in RPMI with 5% FBS at a concentration of 1,000 cells μl^−1^ before loading ~16,000 cells onto the 10x Genomics Chromium platform. Gene expression libraries were prepared using the 10x Genomics Single Cell 3′ Reagent Kits v2 following the user guide (CG00052). The final libraries were loaded on the Illumina HiSeq 4000 sequencing platform using a 28 bp/98 bp read length configuration and targeting a minimum depth of 50,000 reads per cell.

### Computational analysis of the scRNA-seq data

Sequence reads were mapped using 10x Genomics Cell Ranger multi pipeline (cellranger v6.0.0) with the 10x human reference transcriptome (v2020-A). The CellBender ‘remove-background’ tool was applied to the raw Cell Ranger count matrices to eliminate technical artifacts (including ambient RNA)^65^, providing *n* = 34,456 cells. Cells with >500 genes and <5% mitochondrial reads (*n* = 23,011 cells) were retained for downstream analysis. Random downsampling was applied to normalize the median number of unique molecular identifiers (UMIs) per cell between the samples (downsampleMatrix function, DropletUtils R package)^66^. Scrublet^67^ was then used to filter out *n* = 928 doublets (cells with Scrublet score ≥ mean Scrublet score + 2 s.d.), leaving *n* = 22,083 cells for further analysis.

For analyses of the full manifold or separate regions, counts were normalized and log1p-transformed using Scanpy^13^. Highly variable genes (HVGs) were identified per condition (asymptomatic versus symptomatic) using the scanpy.pp.highly_variable_genes function (with batch_key=‘sample_id’ and flavor=‘seurat_v3’ sorting first by ‘highly_variable_nbatches’ and then by ‘highly_variable_rank’). In each case, we retained the union of the top 2,000 HVGs for each condition for downstream analysis. Variation associated with cycle effects (G2M and S phase difference), total UMI number and percentage of mitochondrial counts were regressed out^68^. HVGs were used as input for principal component analysis (PCA), and 20–30 principal components (PCs) were retained based on inspection of the variance ratio plots. Sample integration was performed using the Harmony algorithm^14^.

Clustering analysis of the integrated data was performed with pipeline_scxl.py (https://github.com/sansomlab/tenx)^44^. An exact neighbor graph was computed with Scikit-learn^69^ as implemented in scVelo^70^ (*n* = 20 neighbors, Euclidean distance metric) and used to compute UMAPs and for Leiden clustering across the range of resolutions. Clustering resolutions were compared using the clustree R package^71^. Significant cluster markers were identified (Seurat Findmarkers function, Wilcoxon tests, Benjamini–Hochberg (BH)-adjusted *P* value < 0.05). Initial draft analyses of all cells and the lymphoid and myeloid subregions identified several small clusters of low-quality or likely contaminant cells, which were excluded from the final analysis (*n* = 1,140 cells). For each of the final analyses of the sanitized set of *n* = 20,943 cells, HVGs were rediscovered and the analysis was performed as described. For the final all-cells analysis, 2,709 HVGs were identified, 30 PCs were retained and a clustering resolution of 0.6 was applied, then clusters were combined in ten main cell types and reported in Extended Data Fig. 1b,c. For the final lymphoid cell region analysis, 2,891 HVGs were identified, 30 PCs were retained and a clustering resolution of 0.8 was applied. For the final myeloid cell region analysis, 2,882 HVGs were identified, 30 PCs were retained and a clustering resolution of 0.8 was applied. Clusters were annotated based on automatic cell-type predictions made with the SingleR R package^72^ and manual inspection of the discovered marker genes. To further confirm our cell annotations, we performed (1) reference-based mapping of our cells using the Azimuth R package^18^ and the Azimuth Lung v2 (HLCA)^73–81^ and PBMC reference datasets, and (2) gene set overrepresentation analysis of cluster marker genes using xCell gene sets^17^.

To confirm that our identification of myeloid cell subsets was robust, we used an alternative Seurat-based workflow^24^. For this analysis, normalization was peformed using the sctransform algorithm^23^. HVGs were then identified per condition (asymptomatic versus symptomatic) using Seurat’s FindVariableFeatures function (with selection.method=‘vst’ and nfeatures=2000). Dataset integration was performed (Seurat’s IntegrateData) using a precomputed AnchorSet generated by FindIntegrationAnchors. Finally, PCA and UMAP were calculated, the nearest-neighbor graph was constructed using 30 PCs and clusters were identified using the default Louvain algorithm from the FindClusters function of the Seurat workflow.

Gene set overrepresentation analysis of cluster marker genes was performed using one-sided Fisher exact tests (as implemented in the gsfisher R package, https://github.com/sansomlab/gsfisher) with Gene Ontology (GO) Biological Process (BP), Cellular Component (CC) and Molecular Function (MF); Kyoto Encyclopedia of Genes and Genomes (KEGG) annotations; and xCell gene sets^17^. For this analysis, cluster-specific gene universes were defined as those genes expressed in at least 10% of cells (either within or outside the cluster of interest). BH-adjusted *P* values were computed separately for each ontology.

Macrophages (*n* = 3,628 cells) were extracted from the myeloid region for targeted analyses. Connectivity was assessed using PAGA^39^. Gene set scores (for inflammation, apoptosis and lipid signatures) were computed using the AUCell algorithm^82^. The sets of genes used for this analysis are listed in Supplementary Table 2. RNA velocity of the macrophage clusters was performed with Velocyto^83^ and scVelo^70^ (minimum of 20 unspliced and 20 spliced counts per gene, HVG selection as described above, generalized dynamical model). The CytoTRACE kernel^84^ from the CellRank^85^ Python toolkit was used to perform random walks on a pseudotime transition matrix constructed from a *k*-nearest neighbor (KNN) graph. Differentially expressed genes (DEGs) in TREM2^hi^ versus PLIN^hi^/TREM1^hi^ populations were identified from patient-level pseudobulks (DESeq2 (ref. ^86^) analyis; 547 significant DEGs; paired Wald test, BH-adjusted *P* < 0.1) (Source Data Fig. 3). Gene set enrichment analysis with FGSEA^87^ was performed using the *n* = 15,835 genes tested for differential expression (ranked according to the test statistic) and implementing the multilevel procedure with GO BP, CC and MF and KEGG pathway annotations. To help remove redundant pathways, we applied the FGSEA collapsePathways function (Source Data Fig. 3). BH-adjusted *P* values were calculated separately for each ontology.

For cell–cell communication analysis, NATMI was used (default parameter –interDB lrc2p) to search for possible interactions between literature-supported ligand–receptor pairs from the connectomeDB2020 database^41^. The count matrix was first filtered to remove lowly expressed genes (genes detected in less than ten cells were filtered out). Interactions with a ligand–receptor detection rate > 0.2 and with Edge average expression derived specificity > 0.1 were visualized using custom scripts.

### Reanalysis of community-available scRNA-seq datasets

We reanalyzed the scRNA-seq count matrix from ref. ^32^ for human carotid samples (GSE159677). Cells with >200 genes, <4,000 genes and <10% mitochondrial reads were retained for downstream analysis. Cell barcodes with Scrublet^67^ scores ≥ mean Scrublet score + 2 s.d. were filtered out to exclude likely cell multiplets. Counts were normalized and log1p-transformed using Scanpy^13^. HVGs were identified using the scanpy.pp.highly_variable_genes function (with n_top_genes=3000, flavor=‘seurat_v3’ and batch_key=‘patient’). Variation associated with cycle effects (G2M and S phase difference), total UMI number and percentage of mitochondrial counts were regressed out^68^. HVGs were used as input for integration to remove the batch effect using scVI (batch_key=‘sample_id’ n_latent=30). The neighbor graph and UMAP were computed using Scanpy, then Leiden clustering was implemented across the range of resolutions. For the myeloid analysis, clusters 1, 11 and 12 from the atherosclerotic core were extracted and reanalyzed separately using the same approach (starting from the HVG discovery step).

We also analyzed the count matrix from ref. ^31^ (GSE131780). The raw count matrix was preprocessed in R using Seurat as described previously^9^. A total of 1,889 macrophage barcodes were extracted and reanalyzed using Scanpy. Lowly expressed genes with less than ten counts were removed, and counts were normalized and log1p-transformed. A total of 2,000 HVGs were identified using the scanpy.pp.highly_variable_genes function (flavor=‘seurat_v3’, span=0.5, batch_key=‘Sample’, sorting first by ‘highly_variable_nbatches’ and then by ‘highly_variable_rank’). log1p-transformed values were scaled (max_value=10), PCA was performed and 50 PCs were retained for downstream analysis. Sample integration was performed using the bbknn algorithm. Cells were clustered using the Leiden algorithm (resolution = 1) and visualized using UMAP (minimum distance = 0.1). Reference-based integration with our discovery cohort was performed using scANVI with either dataset as a reference. In both cases, the reference dataset was used to pretrain an scVI model (batch_key=‘sample_id’, n_latent=30, n_layers=2, max_epochs=400). After setting up the scANVI model (max_epochs=20), the query dataset was loaded, and cluster labels were predicted (max_epochs=100).

For murine investigation, six publicly available scRNA-seq datasets from atherosclerotic mouse aortas were reanalyzed (GSE97310 (ref. ^33^), GSE116240 (ref. ^10^), GSE123587 (ref. ^37^), GSE154817 (ref. ^34^), E-MTAB-10743 (ref. ^35^) and GSE135310 (ref. ^36^)). Sequence reads were aligned using the 10x Genomic Cell Ranger multi pipeline (cellranger v6.0.0) and the 10x mouse reference genome mm10 (GENCODE vM23/Ensembl98). High-quality cells with <7.5% mitochondrial reads and >200 genes were retained for downstream analysis. For all downstream analyses, cell counts were normalized and log1p-transformed using Scanpy. HVGs were identified across all samples using the scanpy.pp.highly_variable_genes function (flavor=‘seurat_v3’, sorting first by ‘highly_variable_nbatches’ and then by ‘highly_variable_rank’). For quality control, samples were integrated using the bbknn^88^ algorithm and 3,000 HVGs (batch_key=‘sample_id’). A total of 2,335 doublets were removed using Scrublet. Doublets were identified as cells with Scrublet score > median + 2 s.d. per sample or if >70% of cells within a cluster were identified as doublets. In addition, 606 contaminating nonimmune cells (endothelial cells, fibroblasts, smooth muscle and platelets) were excluded from downstream analysis, resulting in 37,212 immune cells that were reintegrated using scVI^89^ (batch_key=sample_id, n_latent=50) to resolve the *Ptprc* (CD45) landscape. Low-resolution clustering analysis was performed as described above with pipeline_scxl.py (https://github.com/sansomlab/tenx) to identify major cell types (T cells, B cells, neutrophils and MNPs). MNP clusters were identified using singleR (ImmGenData and MouseRNAseqData as reference) and by *Itgam*, *Adgre1*, *Fcgr1* and *Flt3* expression and were subsetted for downstream analysis. Initial draft analysis of MNPs identified 528 contaminating neutrophils and T cells that were removed for the final analysis (final analysis = 33,377 cells). A total of 2,000 HVGs were rediscovered (batch_key=‘study’). Variation associated with total number of UMIs was regressed out using the sc.regress_out function. In Scanpy, log1p-transformed values were scaled (max_value=10), PCA was performed and 50 PCs were retained for downstream analysis. Sample integration was performed using the bbknn algorithm. Cells were clustered using the Leiden algorithm (resolution = 2) and visualized using UMAP (minimum distance = 0.1). The pyorthomap package (https://github.com/vitkl/orthologsBioMART, 10.5281/zenodo.3666961) was used to map gene orthologs between species, and only genes with 1:1 conversions between mouse and human were retained for reference-based integration of both datasets using scANVI^38^. First, the reference dataset was used to pretrain an scVI model (batch_key=‘sample_id’, n_latent=30, n_layers=2, max_epochs=400). After setting up the scANVI model (max_epochs=20), the query dataset with ortholog gene names was loaded, and cluster labels were predicted (max_epochs=100). Gene scores of relevant human gene lists (Supplementary Table 1) in the mouse dataset were calculated using the scanpy.tl.score_genes function after finding mouse 1:1 ortholog gene names using pyorthomap.

### Human plaque bulk RNA-seq from the CPIP

Human carotid plaque samples were obtained from the CPIP biobanks (Lund University, Skåne University Hospital, Malmö, Sweden). Indications for surgery were degree of stenosis >70% (verified by duplex ultrasound) and associated symptoms (stroke, transient ischemic attack or amaurosis fugax) or no symptoms but a degree of stenosis >80%. The study complies with the Declaration of Helsinki, and all patients have provided written informed consent, as previously described^90^. Ethical permission has been obtained from the Lund University review board (reference number 472/2005).

Gene expression of *TREM1*, *PLIN2*, associated cytokines and cell markers^91^ were assessed from the global transcriptome RNA-seq (78 plaques: 51 with symptoms <31 days and 27 without symptoms). Clinical characteristics are presented in Supplementary Table 3. TRIzol was used for RNA extraction, and ribosomal RNA clearing was performed using a Ribo-Zero Magnetic Kit (Epicentre). Strand-specific libraries were prepared with a ScriptSeq v2 RNA-Seq Library Preparation Kit (Epicentre), as previously described^92^. RNA was sequenced using the Illumina HiSeq 2000 and NextSeq platforms. Transcript-level quantification was conducted using Salmon^27^ based on transcriptome release 27 of GENCODE in mapping-based mode. Gene counts were summarized using tximport^28^ and were normalized between samples using a trimmed mean of M values (TMM) by edgeR^93^. Batch effects of sequencing platforms were adjusted by an empirical Bayes method^94^. Finally, gene expression level was shown in log_2_-transformed counts per million (CPM).

An index to identify plaques with a high expression of both *TREM1* and *PLIN2* was generated by dividing plaques in tertiles based on *TREM1* and *PLIN2* gene expression levels. The two generated tertile scores were then added to a summarized score ranging from 2 to 6. Plaques with a combined TREM1/PLIN2 score of 5–6 were considered to be high-expressing plaques, and those with a score of 2–4 were considered to be low-expressing plaques (cut off by median). Gene expression levels of *CCL2*, *CXCL2*, *CXCL3*, *CXCL8*, *TLR2* and *TLR4* were then compared between the two groups using Student’s *t*-test using IBM SPSS Statistics v28 and GraphPad Prism v9.

### Human carotid plaque histology from the CPIP

Carotid plaques from 37 patients were obtained from the CPIP biobank. A 1-mm fragment from the most stenotic region of the plaque was cryosectioned into 8-μm sections for histological analyses. To investigate potential colocalization between PLIN2, TREM1 and TREM2 expression, the frozen plaque tissue sections were fixated in 4% buffered formaldehyde solution (Histolab Products AB) overnight. The tissue was then dehydrated in increasing alcohol concentrations, cleared in xylen and embedded in Histowax (Histolab Products AB). PLIN2 was stained using a primary rabbit anti-human PLIN2 antibody (Sigma, HPA016607) and MACH 3 Rabbit HRP Polymer (Biocare Medical, RP531H). TREM1 was stained using a rabbit anti-human monoclonal TREM1 antibody (Abcam, ab 225861) and a MACH 3 Rabbit HRP Polymer. TREM2 was stained using a rabbit anti-human polyclonal TREM2 antibody (Invitrogen PA5-119690) and a MACH 3 Rabbit HRP Polymer.

To stain neutral lipids, sections were fixed with HistoChoice (Amresco), dipped in 60% isopropanol and then in 0.4% Oil Red O in 60% isopropanol (for 20 min). Macrophages (CD68) were stained using a primary mouse anti-human monoclonal CD68 antibody (DakoCytomation, M0814), diluted to 1:100 in 10% rabbit serum, and a secondary rabbit anti-mouse polyclonal antibody (DakoCytomation, E0413), diluted to 1:200 in 10% rabbit serum. The stained plaque area of each component was analyzed using BioPix iQ v2.1.8.

### Culture of hMDMs

Human peripheral monocytes were isolated from single-donor plateletphoresis residues purchased from the North London Blood Transfusion Service. Peripheral blood mononuclear cells (PBMCs) were isolated by Ficoll-Hypaque centrifugation (specific density, 1.077 g ml^−1^; Sigma-Aldrich, 10771). The monocyte population was enriched by negative selection of unlabeled target cells using a human monocyte enrichment kit (Pan Monocyte Isolation kit, Miltenyi Biotec, 130-096-537), according to the manufacturer´s protocol. The isolated monocytes were cultured in RPMI 10% FBS supplemented with macrophage colony-stimulating factor (M-CSF) for 6 days, after which they underwent specific treatments. Cells were treated with TLR2 ligand FSL-1 (100 ng ml^−1^; InvivoGen, tlr-fsl), TLR4 ligand LPS (1 ng ml^−1^; Enzo Life Sciences, ALX-581-010-L002) or supernatant from ACCM (described above) diluted 2:1, or were left untreated. Cells underwent all treatments for 24 h, after which RNA and intact cells were collected for reverse transcription-quantitative polymerase chain reaction (RT–qPCR) and flow analysis. For the TLR2 knockdown experiments, the following antisense oligonucleotides (LNA-ASOs; Roche Pharma Research and Early Development, RNA Therapeutics Research, Roche Innovation Center Copenhagen, Hørsholm, Denmark, patent number WO2020011869A2) were used: LNA-Ctrl (TGATaagacattTATT) and LNA-TLR2 (TGCttggtttgggaAT), where an uppercase letter represents an LNA nucleoside, LNA C are all 5-methyl C, and a lowercase letter represents a DNA nucleoside and all internucleoside linkages are phosphorothioate internucleoside linkages. Cells were treated at day 3 with either LNA-Ctrl or LNA-TLR2 at a concentration of 10 μM in RPMI 10% FBS with M-CSF until the end of the experiment. At day 6, cells were treated with either TLR2 ligand FSL-1 (100 ng ml^−1^, InvivoGen, tlr-fsl) or supernatant from ACCM, or were left untreated. Cells underwent all treatments for 24 h, after which RNA was collected for RT–qPCR analysis. Data were analyzed using GraphPad Prism v9.

### RNA extraction and RT–qPCR

Total RNA from monocyte-derived macrophages was isolated using an RNeasy Mini Kit (Qiagen, 74106), according to the manufacturer’s instructions. RNA was reverse transcribed (SuperScript II, Invitrogen), and RT–qPCR was performed to quantify relative transcript level using the TaqMan system (Thermo Fisher Scientific): bActin (Hs01060665_g1), TREM1 (Hs00218624_m1), TREM2 (Hs00219132_m1), TLR2 (Hs00610101_m1), TLR4 (Hs01060206_m1), IL-1B (Hs01555410_m1), IL-6 (Hs00174131_m1), PLIN2 (Hs00605340_m1) and CCL2 (Hs00234140).

### Flow cytometry

Around 10^6^ monocyte-derived macrophages per treatment were collected, washed and resuspended in 100 μl of FACS buffer. Cells were labeled with live/dead dye (Invitrogen, L34975) and anti-TREM1 (5 μg per 100 μl; BioLegend, 314906) at 4 °C for 30 min. Then, cells were washed, fixed in cell fix at a dilution of 1:10 (BD CellFIX 34181) for 10 min and resuspended in FACS buffer processed with an LSR II cytometer (BD Biosciences) (gating strategy shown in Supplementary Fig. 4), then analyzed using FlowJo software v10.5.3 (Tree Star Inc.).

### Reporting summary

Further information on research design is available in the Nature Portfolio Reporting Summary linked to this article.

### Supplementary information

Supplementary Information Supplementary Figs. 1–4.

Reporting Summary

Supplementary Tables 1–3 Supplementary Tables 1–3.

### Source data

Source Data Fig. 1 Marker gene summary table and pathway analysis for lymphoid clusters.

Source Data Fig. 2 Marker gene summary table and pathway analysis for myeloid clusters.

Source Data Fig. 3 DESeq2 PLIN2^hi^/TREM1^hi^ versus all other myeloid clusters and pathway analysis for TREM2^hi^ versus PLIN2^hi^/TREM1^hi^.

Source Data Fig. 4 Data for hMDM in vitro analysis.

Source Data Extended Data Fig.10 Data for TREM1 protein expression using flow cytometry.

## Data Availability

The data from the human CPIP cohort presented in this study will be shared in group form, on reasonable request and in compliance with the Swedish General Data Protection Regulation (GDPR) due to data confidentiality of living subjects and ethical and/or legal issues. Requests for data should be directed to I.G. (Isabel.Goncalves@med.lu.se). The time frame for response to requests from the authors is 4 weeks. Requesters will be required to sign a data access agreement to ensure the appropriate use of the study data. The scRNA-seq data that support the findings of this study have been deposited in the National Center for Biotechnology Information (NCBI) Gene Expression Omnibus (GEO) under the accession code GSE210152. All other data supporting the findings in this study and included in the main article and associated files were downloaded from GEO (https://www.ncbi.nlm.nih.gov/geo) and the European Molecular Biology Laboratory (EMBL) European Bioinformatics Institute (EBI) (https://www.ebi.ac.uk). For murine data, the following datasets were downloaded: GSE97310 (ref. ^33^), GSE116240 (ref. ^10^), GSE123587 (ref. ^37^), GSE154817 (ref. ^34^), E-MTAB-10743 (ref. ^35^) and GSE135310 (ref. ^36^). For human analysis, the following datasets were downloaded: GSE131780 (ref. ^31^) and GSE159677 (ref. ^32^).

## Code availability

Analysis of single-cell data was performed using ‘tenx’ bioinformatic pipelines (https://github.com/sansomlab/tenx). Gene set overrepresentation analysis of cluster marker genes was performed using the gsfisher R package (https://github.com/sansomlab/gsfisher).
