# Author Correction: Lipid-associated macrophages transition to an inflammatory state in human atherosclerosis, increasing the risk of cerebrovascular complications

**DOI:** 10.1038/s44161-023-00328-5

**Published:** 2023-08-07

**Authors:** Lea Dib, Lada A. Koneva, Andreas Edsfeldt, Yasemin-Xiomara Zurke, Jiangming Sun, Mihaela Nitulescu, Moustafa Attar, Esther Lutgens, Steffen Schmidt, Marie W. Lindholm, Robin P. Choudhury, Ismail Cassimjee, Regent Lee, Ashok Handa, Isabel Goncalves, Stephen N. Sansom, Claudia Monaco

**Affiliations:** 1 Kennedy Institute of Rheumatology, Nuffield Department of Orthopaedics, Rheumatology and Musculoskeletal Sciences, University of Oxford, Oxford, UK; 2 Department of Clinical Sciences Malmö, Clinical Research Center, Lund University, Malmö, Sweden; 3 Department of Cardiology, Skåne University Hospital, Malmö, Sweden; 4 Wallenberg Center for Molecular Medicine, Lund University, Lund, Sweden; 5 Cardiovascular Medicine and Immunology, Mayo Clinic, Rochester, MN USA; 6 Roche Pharma Research and Early Development, RNA Therapeutics Research, Roche Innovation Center Copenhagen, Hørsholm, Denmark; 7 Radcliffe Department of Medicine, University of Oxford, Oxford, UK; 8 Nuffield Department of Surgical Sciences, University of Oxford, Oxford, UK

**Keywords:** Cardiovascular biology, Inflammation

Correction to: *Nature Cardiovascular Research* 10.1038/s44161-023-00295-x. Published online 26 June 2023.

This paper was originally published under a standard Springer Nature license (© The Author(s), under exclusive licence to Springer Nature Limited). It is now available as an open-access paper under a Creative Commons Attribution 4.0 International license, © The Author(s). The error has been corrected in the online version of the article.

