# Supplementary material for: Lipid-associated macrophages transition to an inflammatory state in human atherosclerosis, increasing the risk of cerebrovascular complications
Source: Nat Cardiovasc Res. 2023 Jun 26;2(7):656–72. doi: 10.1038/s44161-023-00295-x (PMC7615632; doi:10.1038/s44161-023-00295-x)

nature cardiovascular research

Article https://doi.org/10.1038/s44161-023-00295-x

Lipid-associated macrophages transition to an inflammatory state in human atherosclerosis, increasing the risk of cerebrovascular complications

In the format provided by the authors and unedited

Supplementary Figure 1. Quality control analysis of the Discovery cohort single cell data. The bar plots show per sample sample QC summary statistics following mapping and quantitation with CellRanger (**a**). The volcano plots show the distribution of the number genes (left), percent mitochondrial reads (middle) and total number UMIs per sample as computed after using CellBender 'remove-background' tool (**b**).

Supplementary Figure 1

a.

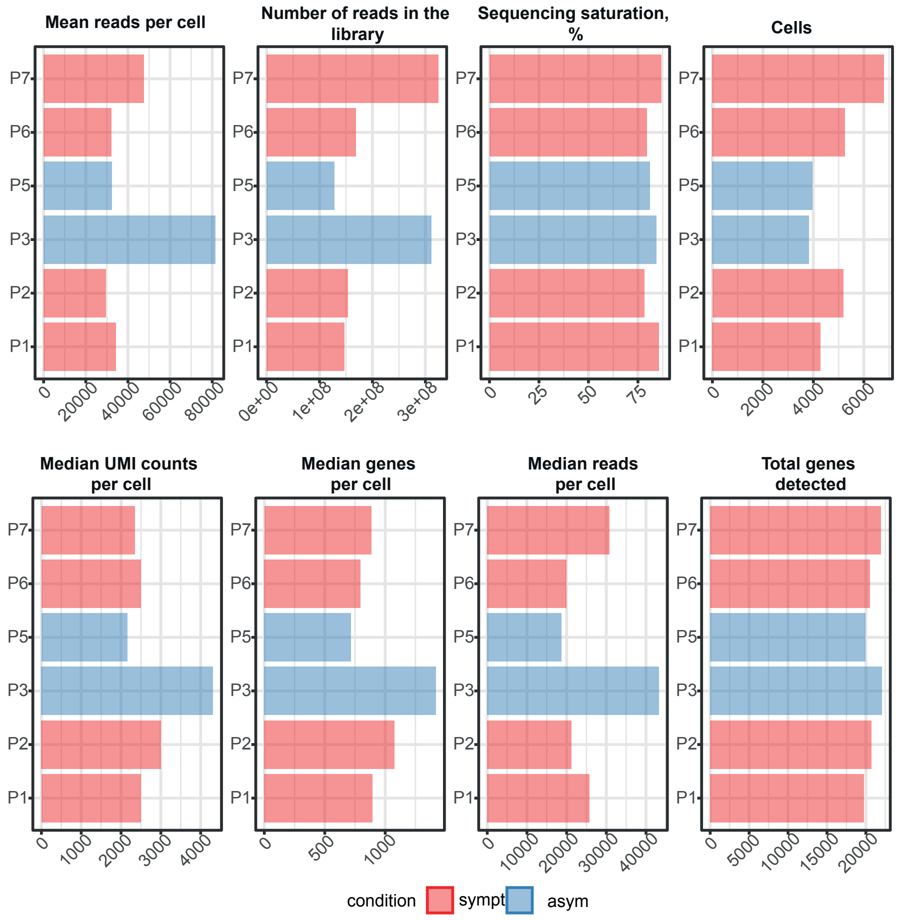

b.

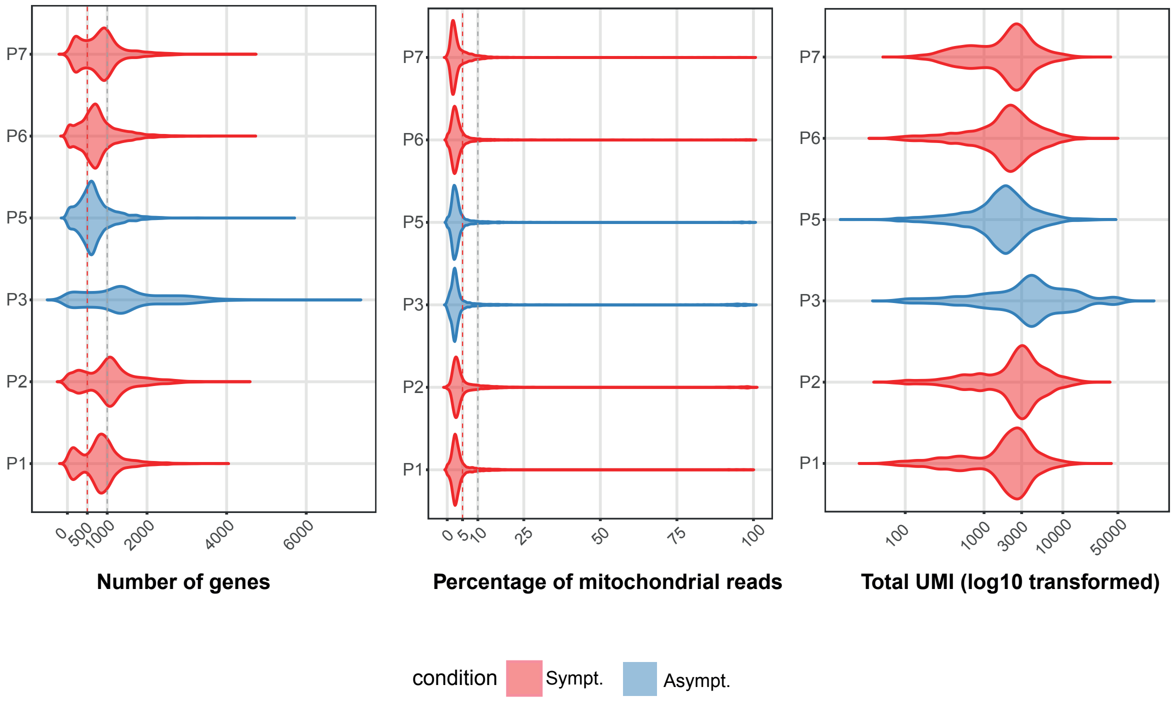

Supplementary Figure 2. Re-analysis of published human carotid atherosclerosis scRNA seq data from Wirka et al. (GSE131780). The myeloid analysis generated 11 clusters (**a**), markers genes of interest were plotted on UMAP (**b**) and alluvial plot projecting Wirka clusters on our myeloid population and vice versa is shown (**c**). The majority of the macrophages from this study had a phenotype similar to our C1Q cluster, likely due to inclusion of the coronary adventitia in this study.

Supplementary Figure 2

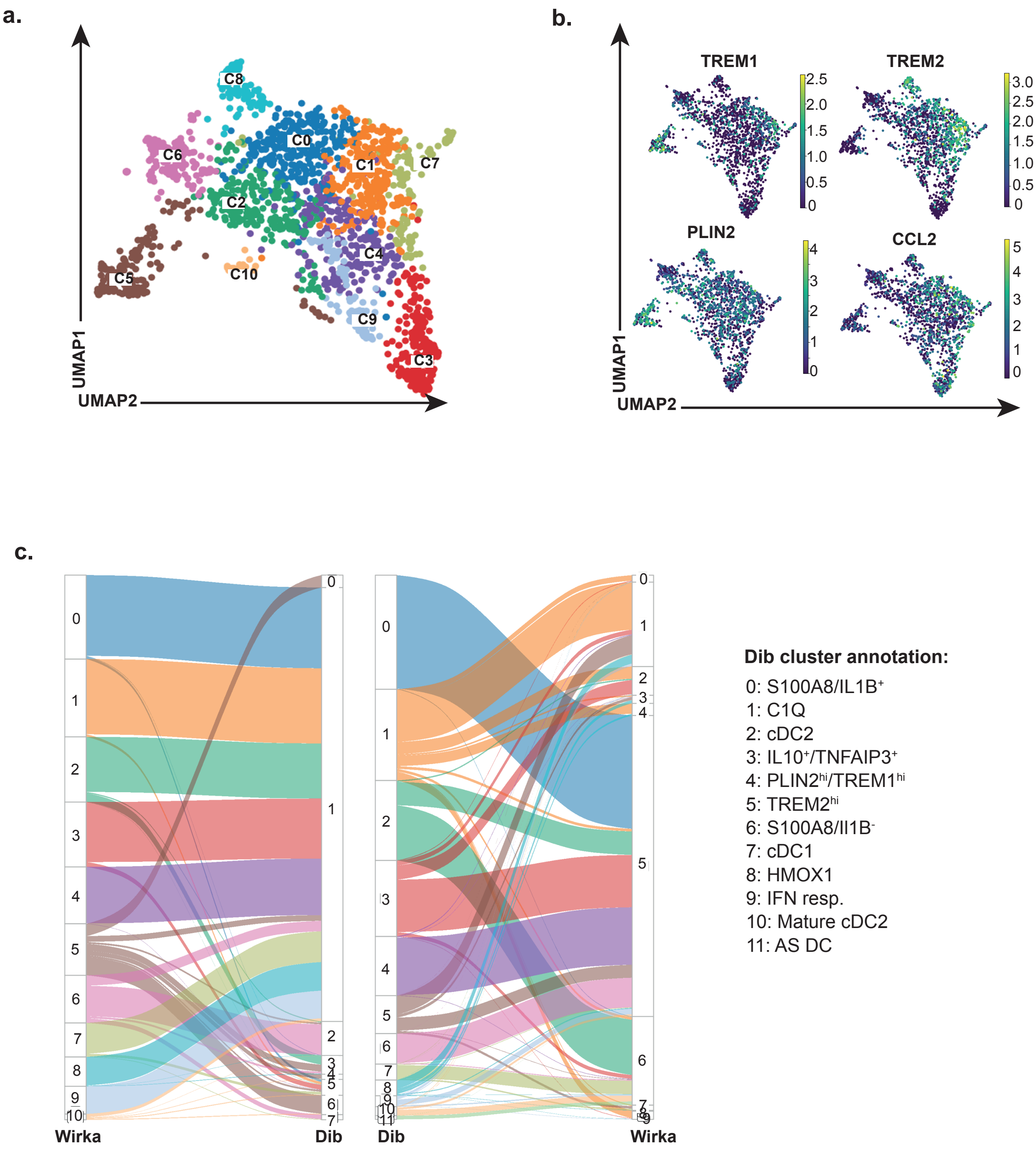

Supplementary Figure 3. Cross-species analysis of human coronary atherosclerosis and murine atherosclerosis scRNA seq studies.

We constructed an integrated myeloid cell atlas using six available murine scRNA-seq datasets and compared the identified clusters to those from human discovery cohort using label transfer approach with the scVI algorithm[89]. The UMAP shows the integrated atlas of myeloid cells from the six murine studies (**a). **The UMAPs show the cells faceted by study **(b**). Visualisation of selected markers on the UMAP shows distinct Macrophage (Adgre1+) populations, of which 2 are Trem2+, monocyte clusters (Ly6c2^{+} and Ly6c2^{-}) and DC clusters (Ftl3^{+}) (**c**). The human PLIN2^{hi}/TREM1^{hi} did not have a clear analog in the murine data. As an alternative approach to identify a PLIN2^{hi}/TREM1^{hi} subset in the mouse data we computed 'Trem1vstrem2' and 'trem2vstrem1' scores (PLIN2^{hi}/TREM1^{hi} subset gene score and TREM2^{hi} subset score respectively) using the mouse orthologs of genes differentially expressed between these clusters in our human data (see methods) and plotted these scores on the integrated murine UMAP. The 'Trem1vstrem2' score was highest in the murine monocyte clusters (**d**) whereas the 'trem2vtrem1' score mapped to the murine Trem2 populations (**e**). Sorted CD64^{+} foamy macrophages from Kim et al. confirms that murine foam cells show no iLAM signature where they have minimal Trem1vstrem2 score (**f**) and high trem2vtrem1 score signature (**g**)**.** Alluvial plots alluvial plots to show the projection of cells from the murine integrated analysis (Mouse Integ) onto our human cluster annotations (Dib.) and vice versa (**h).**

Supplementary Figure 3

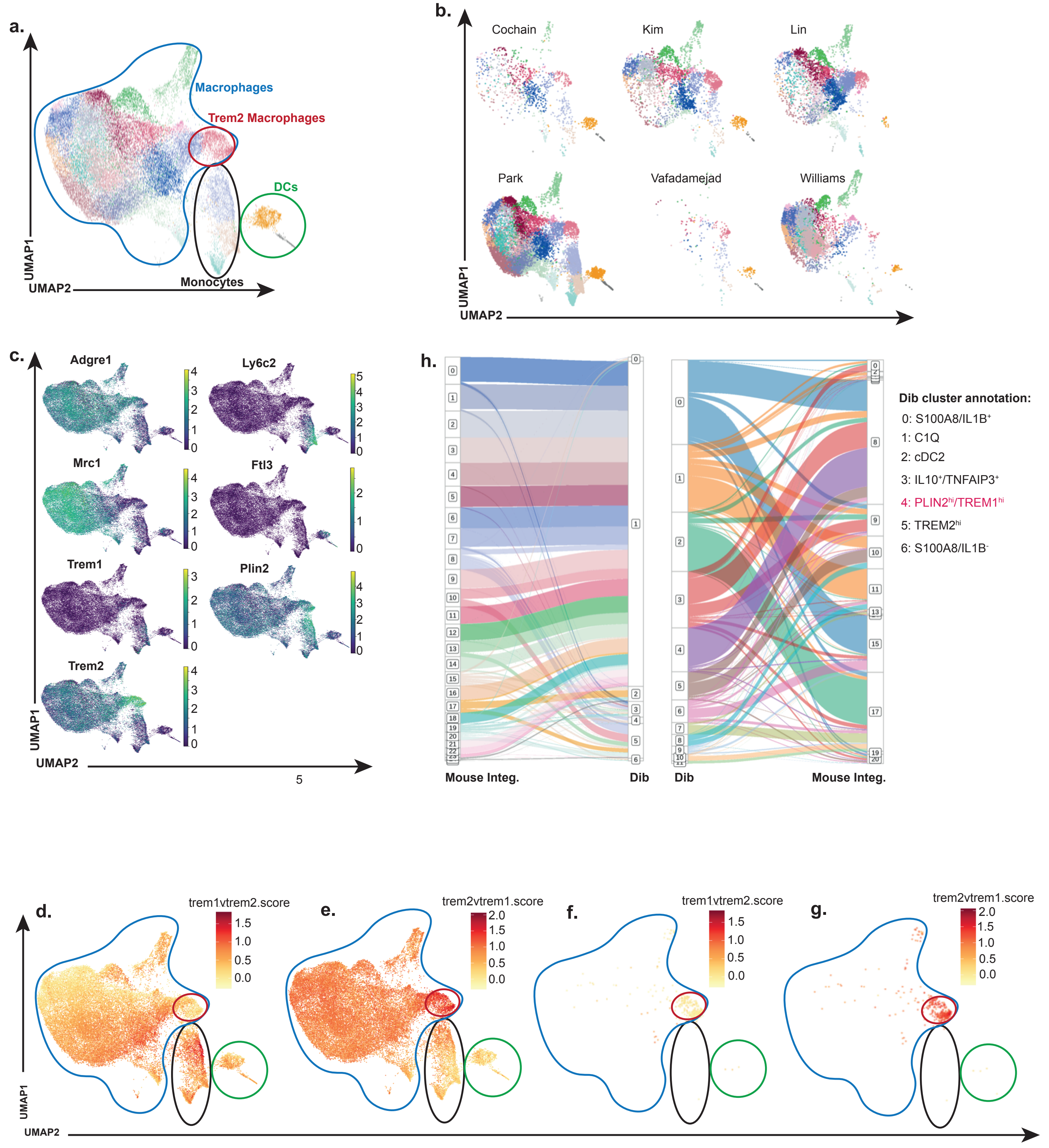

Supplementary Figure 4. Gating strategy for populations of interest. For the scRNAseq studies, carotid plaques were enzymatically digested stained with Live/Dead dye (APC-Cy7A) and anti-CD45 (FITC) FACS ARIAIII (BD Biosciences). Gating strategy consisted of: **(a)** removing dead cells using Live/Dead dye (APC-Cy7), **(b)** gating on CD45+ cells (FITC), **(c)** gating on FSC/SSC to remove debris and (**d)** selecting single cells using FSC-A/FSC-W and SSC-A/SSC-H**.** For in vitro, human monocyte-derived macrophages, cells were gated on (**e**) FSC/SSC to remove debris and (**f**) APC-Cy7A (Live/Dead dye) negative area to gate on live cells.

Supplementary Figure 4

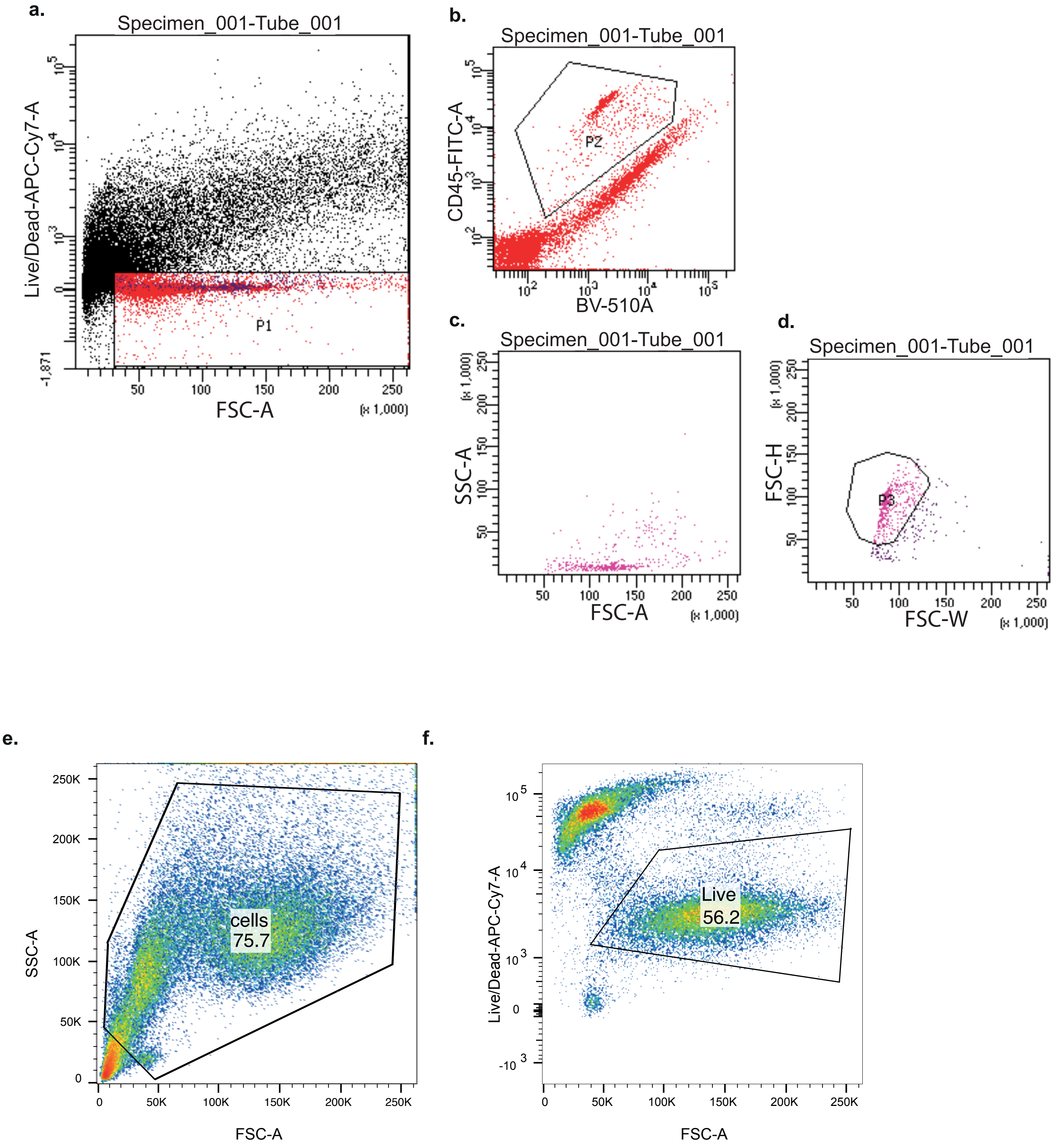

Supplement: Supplementary file 1 — Supplementary Figs. 1–4. [file 44161_2023_295_MOESM1_ESM.pdf]
