# Supplementary material for: Lipid-associated macrophages transition to an inflammatory state in human atherosclerosis, increasing the risk of cerebrovascular complications
Source: Nat Cardiovasc Res. 2023 Jun 26;2(7):656–72. doi: 10.1038/s44161-023-00295-x (PMC7615632; doi:10.1038/s44161-023-00295-x)
Supplement: Supplementary file 2 — Reporting Summary [file 44161_2023_295_MOESM2_ESM.pdf]

nature portfolio

Corresponding author(s): Claudia Monaco & Stephen Sansom

Last updated by author(s): May 22, 2023

Reporting Summary

Nature Portfolio wishes to improve the reproducibility of the work that we publish. This form provides structure for consistency and transparency in reporting. For further information on Nature Portfolio policies, see our Editorial Policies and the Editorial Policy Checklist.

Statistics

For all statistical analyses, confirm that the following items are present in the figure legend, table legend, main text, or Methods section.

n/a	Confirmed	
☐	☒	The exact sample size (*n*) for each experimental group/condition, given as a discrete number and unit of measurement
☐	☒	A statement on whether measurements were taken from distinct samples or whether the same sample was measured repeatedly
☐	☒	The statistical test(s) used AND whether they are one- or two-sided<br>*Only common tests should be described solely by name; describe more complex techniques in the Methods section.*
☐	☒	A description of all covariates tested
☐	☒	A description of any assumptions or corrections, such as tests of normality and adjustment for multiple comparisons
☐	☒	A full description of the statistical parameters including central tendency (e.g. means) or other basic estimates (e.g. regression coefficient) AND variation (e.g. standard deviation) or associated estimates of uncertainty (e.g. confidence intervals)
☐	☒	For null hypothesis testing, the test statistic (e.g. F, t, r) with confidence intervals, effect sizes, degrees of freedom and P value noted<br>*Give P values as exact values whenever suitable.*
☒	☐	For Bayesian analysis, information on the choice of priors and Markov chain Monte Carlo settings
☒	☐	For hierarchical and complex designs, identification of the appropriate level for tests and full reporting of outcomes
☒	☐	Estimates of effect sizes (e.g. Cohen's *d*, Pearson's *r*), indicating how they were calculated

Our web collection on statistics for biologists contains articles on many of the points above.

Software and code

Policy information about availability of computer code

Data collection	Single cell RNA sequencing data was collected using the 10xChromium System and CellRanger multi pipeline (version cellranger-6.0.0) with the 10x human reference transcriptome (version 2020-A). RNA was sequenced using the Illumina HiSeq2000 and the NextSeq platforms. Flow cytometry was performed using LSRII cytometer (BD Biosciences) and cell sorting was performed using FACS ARIAIII (BD Biosciences). Analysis was performed using FlowJo 10.5.3.
Data analysis	For scRNA seq, sequence reads were mapped using 10x Genomics CellRanger multi pipeline (version cellranger-6.0.0) with the 10x human reference transcriptome (version 2020-A). The CellBender 'remove-background' tool (https:// github.com/broadinstitute/CellBender), random down-sampling (https://bioconductor.org/packages/DropletUtils) and Scrublet (https://github.com/AllonKleinLab/scrublet) were used. Sample integration was performed using the harmony algorithm(https://github.com/immunogenomics/harmony). Clustering analysis of the integrated data was performed with pipeline_scxl.py (https://github.com/sansomlab/tenx). Gene set over-representation analysis of cluster marker genes was performed using one-sided Fisher's exact tests (as implemented in the 'gsfisher' R package https://github.com/sansomlab/gsfisher/) with GO Biological Process (BP), Cellular Component (CC) and Molecular Function (MF) and KEGG annotations.<br>Graphpad prism 9 and IBM SPSS Statistics v.28 were used for statistical analysis. IHC quantification was analysed using Biopix iQ 2.1.8 .

For manuscripts utilizing custom algorithms or software that are central to the research but not yet described in published literature, software must be made available to editors and reviewers. We strongly encourage code deposition in a community repository (e.g. GitHub). See the Nature Portfolio guidelines for submitting code & software for further information.

Data

Policy information about availability of data

All manuscripts must include a data availability statement. This statement should provide the following information, where applicable:

- Accession codes, unique identifiers, or web links for publicly available datasets
- A description of any restrictions on data availability
- For clinical datasets or third party data, please ensure that the statement adheres to our policy

The data from the human CPIP cohort presented in this study will be shared in group form, on reasonable request and in compliance with the Swedish GDPR regulations due to data confidentiality of living subjects and ethical/legal issues. Requests should be directed to I.G. (Isabel.Goncalves@med.lu.se). The timeframe for response to requests from the authors is 4 weeks. Requestors will be required to sign a data access agreement to ensure the appropriate use of the study data. The single-cell RNA-sequencing data that support the findings of this study have been deposited in the NCBI Gene Expression Omnibus (GEO) with the accession code GSE210152.

Research involving human participants, their data, or biological material

Policy information about studies with human participants or human data. See also policy information about sex, gender (identity/presentation), and sexual orientation and race, ethnicity and racism.

Reporting on sex and gender	Sex was categorized as self reported by patients. Experimental design did not specify sex stratification because patients were recruited according to surgery eligibility and inclusion criteria. No clear effect of sex on symptomatic vs asymptomatic disease was found.
Reporting on race, ethnicity, or other socially relevant groupings	No information pertaining to social or ethnic background of the human participants was disclosed in the current manuscript.
Population characteristics	Patients characteristics and covariates are reported in Suppl.Table 2. Analysis between symptomatic and asymptomatic was performed for all covariates.
Recruitment	For the Discovery cohort, patients undergoing carotid endarterectomy surgery gave their written informed consent to have their discarded and anonymised plaque tissue collected as part of the Oxford Peripheral Vascular Disease Study (OxPVD). For The Carotid Plaque Imaging Project (CPIP) (Validation cohort), patients eligible for carotid endarterectomy were enrolled and gave their written consent forms for tissue and data collection.
Ethics oversight	OxPVD Study (Discovery cohort) was approved by UK National Research Ethics Services (RREC2989 and RREC08/H0706/129). The CPIP study (Validation cohort) was approved by the Lund University review board (reference number 472/2005 and 2014/904).

Note that full information on the approval of the study protocol must also be provided in the manuscript.

Field-specific reporting

Please select the one below that is the best fit for your research. If you are not sure, read the appropriate sections before making your selection.

☒ Life sciences ☐ Behavioural & social sciences ☐ Ecological, evolutionary & environmental sciences

For a reference copy of the document with all sections, see nature.com/documents/nr-reporting-summary-flat.pdf

Life sciences study design

All studies must disclose on these points even when the disclosure is negative.

Sample size	We did not perform sample size calculation for this study. Sample size was determined by tissue availability and size.
Data exclusions	One sample from the discovery cohort (scRNA seq) was excluded due to poor sequencing quality which included low mean read/cell and total read and low sequencing saturation.
Replication	Our discovery cohort consisted of 6 patients, we identified in our single cell analysis similar populations in all of the studied patients showing a high reproducibility of the findings. In addition, we validated our main findings from the discovery cohort using a larger scale Validation cohort (CPIP). The cluster of interest found in scRNAseq data was identified in the validation cohort. Induction of the transcriptional signature of this cluster was also duplicated in vitro.
Randomization	Randomization is not applicable. Specimen were collected from all patients undergoing carotid endarterectomy as they were made available.
Blinding	Researchers processing the carotid samples were blinded. Plaque quantification analyses were performed blindly.

Reporting for specific materials, systems and methods

We require information from authors about some types of materials, experimental systems and methods used in many studies. Here, indicate whether each material, system or method listed is relevant to your study. If you are not sure if a list item applies to your research, read the appropriate section before selecting a response.

Materials & experimental systems

n/a	Involved in the study
☐	☒ Antibodies
☒	☐ Eukaryotic cell lines
☒	☐ Palaeontology and archaeology
☒	☐ Animals and other organisms
☒	☐ Clinical data
☒	☐ Dual use research of concern
☒	☐ Plants

Methods

n/a	Involved in the study
☒	☐ ChIP-seq
☐	☒ Flow cytometry
☒	☐ MRI-based neuroimaging

Antibodies

Antibodies used

Anti-human antibodies were used:
Anti-human CD45-FITC: Clone HI30, Cat.No. 304006, Company Biolegend, Dilution 5:100 (5ug/100ul)
Anti-human TREM1-PE: Clone TREM-26, Cat.No. 314906, Company Biolegend, Dilution 5:100 (5ug/100ul)
Anti-human CD68 (unconjugated): Clone KP1, Cat.No. M0814, Company DakoCytomation, Dilution 1:100
Anti-human PLIN2 (unconjugated): Polyclonal, Cat.No. HPA016607, Company Sigma Aldrich, Dilution 1:200 (1ul/ml)
Anti-human TREM1 (unconjugated): Clone EPR22060-229, Cat.No. ab225861, Company Abcam, Dilution 2ug/ml
Anti-human TREM2 (unconjugated), Polyclonal, Cat.No.PA5-119690, Company Invitrogen, Dilution 2ug/ml
Secondary antibody polyclonal rabbit anti-mouse, Cat no. E0413, Company (DakoCytomation, Glostrup, Denmark), Dilution 1:200
MACH3 (HRP-polymer), Cat.No RP531H, Company Biocare Medical, Ready to use dilution

Validation

All antibodies underwent significant quality control by their respective manufacturers.
For Biolegend antibodies (CD45 and TREM1) and as stated on their websites (https://www.biolegend.com/en-us/quality/quality-control), flow cytometry antibodies are validated as follows 1) specificity testing of 1-3 target cell types with either single- or multi-color analysis (including positive and negative cell types) 2) once specificity is confirmed, each new lot must perform with similar intensity to the in-date reference lot. Brightness (MFI) is evaluated from both positive and negative populations 3) each lot product is validated by QC testing with a series of titration dilutions.
For PLIN2 antibody, it is a Prestige Antibody powered by Atlas Antibodies and extensively validated by the Human Protein Atlas (HPA) project. Every Prestige Antibody is tested in the following ways: 1) IHC tissue array of 44 normal human tissues and 20 of the most common cancer type tissues 2) Protein array of 364 human recombinant protein fragments (https://www.sigmaaldrich.com/GB/en/product/sigma/hpa016607).
For CD68 Dako antibody, was validated through SDS-PAGE analysis of immunoprecipitates formed between the antibody and 125I-labeled lysates from human spleen with B-cell lymphoma rich in macrophages).
For CellSignaling antibody (TREM2) and as stated in their website (https://www.cellsignal.com/about-us/cst-antibody-validation-principles), the company adhere to the Hallmarks of Antibody Validation™, six complementary strategies that can be used to determine the functionality, specificity, and sensitivity of an antibody in any given assay as published in Nature Methods (2016).

Flow Cytometry

Plots

Confirm that:

☒ The axis labels state the marker and fluorochrome used (e.g. CD4-FITC).

☒ The axis scales are clearly visible. Include numbers along axes only for bottom left plot of group (a 'group' is an analysis of identical markers).

☒ All plots are contour plots with outliers or pseudocolor plots.

☒ A numerical value for number of cells or percentage (with statistics) is provided.

Methodology

Sample preparation

For scRNAseq samples: Cell suspensions from freshly digested carotid plaques were obtained as follows: the tissue was extensively washed in RPMI, finely minced and tissue fragments were incubated in collagenase type I (400 units/ml, Sigma C9722), elastase type III (5 units/ml, Worthington, LS006365), and DNase (300 units/ml, Sigma D5025), with 1 mg/ml soybean trypsin inhibitor (Sigma T6522), 2.5 µg/ml polymixin B (Sigma P4932), and 2 mM CaCl2, in RPMI medium 1640 with 5% FBS in a shaker at 37°C for 45 min. Cell suspension was filtered through an 80-µm Nylon mesh and washed in media. Collected cells were incubated with live/dead stain at 1:1,000 in PBS for 10min at 4°C. Cells were then washed with FACS buffer (PBS with

2% FBS), stained for CD45 antibody for 30 min at RT then washed twice with FACS buffer, resuspended in a final FACS buffer volume of 500µl, filtered through an Easy Strain 100-µm cell strainer and sorted for live CD45+ cells using FACS ARIAIII (BD Biosciences).

For in vitro studies: Human peripheral monocytes were isolated from single-donor plateletphoresis residues. Peripheral blood mononuclear cells (PBMC) were isolated by Ficoll/Hypaque centrifugation. The monocyte population was enriched by negative selection of unlabeled target cells using a human monocyte enrichment kit. The isolated monocytes were cultured in RPMI 10%FBS supplemented with M-CSF for 6 days after which they underwent specific treatments. Cells were treated with either TLR2 ligand, FSL-1 (100ng/ml); TLR4 ligand LPS (1ng/ml); supernatant from atheroma culture diluted 2:1 or left untreated. Cells underwent all treatments for 24 hrs after which intact cells were collected for flow analysis. Around 1 million monocyte-derived macrophages/treatment were collected, washed and resuspended in 100ul FACS buffer. Cells were labelled with live/dead dye and anti-TREM1 for 30 min at 4ºC. After which cells were washed, fixed in cell fix at 1:10 dilution for 10 minutes and resuspended in FACS buffer to be processed.

Instrument	LSRII cytometer (BD Biosciences) and FACS ARIAIII (BD Biosciences)
Software	FlowJo software (Tree Star Inc.).
Cell population abundance	Live CD45+ cells were all used for scRNAseq and all cells were ran through the 10x chromium platform. Analysis showed minimal contamination of CD45- cells.
Gating strategy	Dead cells were removed using Live/Dead dye and live CD45+ cells were gated. Cells were gated on FSC/SSC to remove debris. Single cells were selected using FSC-A/FSC-W and SSC-A/SSC-H. For in vitro studies, cells were gated on FSC/SSC to remove debris, dead cells were removed using Aqua dye.

☒ Tick this box to confirm that a figure exemplifying the gating strategy is provided in the Supplementary Information.
